# Conjugated Polymers Containing BODIPY and Fluorene Units for Sensitive Detection of CN^−^ Ions: Site-Selective Synthesis, Photo-Physical and Electrochemical Properties

**DOI:** 10.3390/polym9100512

**Published:** 2017-10-14

**Authors:** Tian He, Danting Tang, Cuiling Lin, Xi Shen, Chenjie Lu, Luonan Xu, Zhengye Gu, Zheng Xu, Huayu Qiu, Qian Zhang, Shouchun Yin

**Affiliations:** College of Material Chemistry and Chemical Engineering, Hangzhou Normal University, Hangzhou 310036, China; hetian81@163.com (T.H.); DeniseTDT@163.com (D.T.); 15888757435@126.com (C.Li.); shenxi0616@163.com (X.S.); 15868140472@139.com (C.Lu); xuluonan@126.com (L.X.); guzhengye@126.com (Z.G.); zhengxu@hznu.edu.cn (Z.X.); hyqiu@hznu.edu.cn (H.Q.)

**Keywords:** BODIPY, conjugated copolymers, site-selective, cyanide anion

## Abstract

Conjugated polymers containing distinct molecular units are expected to be very interesting because of their unique properties endowed by these units and the formed conjugated polymers. Herein, four new conjugated copolymers based on fluorene and 4,4’-difluoro-4-bora-3a,4a-diaza-*s*-indacene (BODIPY) have been designed and synthesized via Sonogashira polymerization. The fluorene unit was attached to the 3,5- or 2,6-positions of BODIPY by ethynylenes or *p*-diacetylenebenzene. The obtained polymers show good thermal stability and broad absorption in the wavelength range from 300 to 750 nm. The effects of site-selective copolymerization and conjugation length along the polymer backbone on the optoelectronic and electrochemical properties of these copolymers were systematically studied by UV-Vis spectroscopy, photoluminescence (PL) and cyclic voltammetry. Besides, it is found that the BODIPY-based copolymers exhibit selectively sensitive responses to cyanide anions, resulting in obvious change of UV-Vis absorption spectra and significant fluorescence quenching of the polymers among various common anions.

## 1. Introduction

4,4-Difluoro-4-bora-3a,4a-diaza-s-indacene (BODIPY) derivatives have been widely investigated in the fields of chemical sensors, solar cells, biological labeling, photodynamic therapy and nonlinear optical materials because of its unique advantages of high chemical and photo-stability, high extinction coefficients and fluorescence quantum yields, and narrow absorption and emission bands [1,2,3,4,5,6,7,8,9,10,11]. More interestingly, its optical properties could be effectively tuned by functionalizing BODIPY cores at the 2,6-, or 3,5-positions, or fusing some aromatic rings to the BODIPY core [1,2,3,4,5,12]. Incorporation of BODIPY dyes into the backbone of conjugated polymers is expected to integrate the unique properties of both BODIPY and conjugated polymers into one material for various potential applications, but the reports are limited [3,13,14,15,16,17,18]. Besides, much less attention has been paid to studying the effects of site-selective polymerization on their optoelectronic properties [19,20].

Polyfluorene and its derivatives have been distinguishing light-emitting materials owing to their highly efficient photoluminescence and electroluminescence, excellent thermal and chemical stability, good solubility and film-forming properties [21,22,23,24,25,26,27,28]. Previous studies demonstrated that incorporation of suitable narrow bandgap chromophores into the side chain or main chain of wide bandgap polyfluorene hosts could realize red and green emitters [25,29]. Based on these, incorporation of BODIPY into polyfluorene backbone is expected to obtain a fluorescent conjugated polymer with excellent optical properties.

**Scheme 1:**
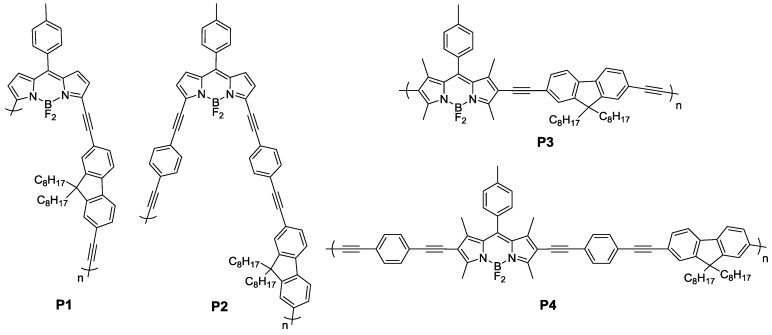
Chemical structures of polymers P1, P2, P3 and P4.

Herein, we have designed and synthesized four novel BODIPY-fluorene alternating copolymers (Scheme 1). Polymers P1 and P3 were synthesized by the Sonogashira cross-coupling reaction in, which fluorenes were attached to the 2,6- or 3,5- positions of BODIPY units through an ethynylene bridge, respectively. For comparison, polymers P2 and P4 with fluorene and BODIPY connected through a *p*-diacetylenebenzene bridge were also obtained. The effect of different connectivity on the BODIPY cores and conjugation length along the polymer backbone on the optoelectronic and electrochemical properties of these copolymers was systematically investigated. Interestingly, these copolymers could selectively exhibit response to CN^−^, as demonstrated by the significant change of UV-Vis absorption and fluorescence spectra among various common anions.

## 2. Materials and Methods

### 2.1. Materials

Unless otherwise stated, all reagents were purchased from commercial suppliers, and used without further purification. 1-ethynyl-4-(trimethylsilylethynyl)benzene, 4,4'-difluoro-3,5-dibromo-8-(4-tolyl)-4-bora-3a,4a-diaza-*s*-indacene (M1), 2,7-ethynyl-9,9-dioctylfluorene (M1’), 4,4’-difluoro-1,3,5,7-tetramethyl-8-(4-tolyl)-4-bora-3a,4a-diaza-*s*-indacene (M-BODIPY) and 4,4’-difluoro-2,6-diiodo-1,3,5,7-tetramethyl-8-(4-tolyl)-4-bora-3a,4a-diaza-*s*-indacene (M3) were synthesized according to the literature [20,30]. Anhydrous tetrahydrofuran (THF) was distilled from sodium/benzophenoneketyl prior to use. Triethylamine (Et_3_N) was distilled from potassium hydroxide prior to use.

### 2.2. Instruments

^1^H NMR spectra (500 MHz) in CDCl_3_ were recorded on a Bruker Advance 500 (Bruker, Fällanden Switzerland) at 298 K and referenced to tetramethylsilane (0.00 ppm) as an internal standard. ^13^C NMR spectra (125 MHz) in CDCl_3_ were referenced to the CDCl_3_ (77.16 ppm) signal. Mass spectra were recorded on a Hewlett-Packard 5989 A mass spectrometer (Hewlett-Packard, Palo Alto, CA, USA) perated in electrospray ionization (ESI) mode. High-resolution mass data were obtained with a MS50TC instrument (Kratos, Manchester, UK). Ultraviolet–Visible (UV-Vis) absorption spectra were recorded on a Lambda 750 UV-Vis spectrophotometer (PerkinElmer, Waltham, MA, USA), and fluorescence spectra were recorded on a F-7000 spectrofluorometer (HITACHI, Tokyo, Japan). Fourier transform infrared (FT-IR) spectra were recorded on a Nicolet 5700 spectrometer (Thermo fisher, Waltham, MA, USA) in the 400–4000 cm^−1^ region. The molecular weights of the polymers were determined using polystyrene as the standard by gel permeation chromatography (GPC) with a Waters Associates liquid chromatograph (Waters, Milford, MA, USA) equipped with a Waters 515 pump, a Wyatt injector with a stand kit, a CBL Model 200 column heater, a Wyatt Optilab T-rEx differential refractometer. All polymers were firstly dissolved in THF (ca. 4 mg/mL), and, before being injected into the GPC system, the solutions were filtered through 0.45 μm Poly tetra-fluoroethylene (PTFE) syringe-type filters. THF was the eluent at a flow rate of 1.0 mL min^−1^. The column temperature was kept at 35 °C. Cyclic voltammetry (CV) were performed on a LK2005A electrochemical work station (Lanlike, Tianjin, China) at a scan rate of 100 mV s^−1^. All CV measurements were carried out in dry THF with tetrabutylammonium phosphorus hexafluoride (Bu_4_NPF_6_, 0.1 M) as the supporting electrolyte at room temperature and using a conventional three-electrode configuration with a glassy carbon electrode as the working electrode, a saturated calomel electrode (SCE) as the reference electrode, and a Pt wire as the counter electrode.

### 2.3. Synthesis

#### 2.3.1. Synthesis of Monomers

4,4’-difluoro-3,5-(4-(trimethylsilylethynyl)phenylethynyl)-8-(4-tolyl)-4-bora-3a,4a-diaza-*s*-indacene (1): 4,4’-difluoro-3,5-dibromo-8-(4-tolyl)-4-bora-3a,4a-diaza-*s*-indacene (438 mg, 1 mmol) and 1-ethynyl-4-(trimethylsilylethynyl)benzene (495 mg, 2.5 mmol) were dissolved in the mixture of THF (150 mL) and Et_3_N (800 μL) under argon. Then PdCl_2_(PPh_3_)_2_ (35 mg, 0.05 mmol) and CuI (10 mg, 0.05 mmol) were added to this solution. After stirring at 60 °C for 10 h, the reaction mixture was poured into water (50 mL) and extracted with CH_2_Cl_2_ (3 × 50 mL). The organic layer was washed with water for twice and dried over anhydrous Na_2_SO_4_ for 3 h. The solvent was removed by evaporation under reduced pressure. Then the crude product was purified by silica gel column chromatography using petroleum ether/CH_2_Cl_2_ (2:1, *v/v*) as eluent to give blue solid 1 (480 mg, 50.4% yield). FT-IR (KBr), *υ* (cm^–1^): 2924, 2854 (CH_3_), 2201, 2156 (C≡C), 1609, 1588, 1410, 862 (Ar). ^1^H NMR (500 MHz, CDCl_3_) δ (ppm): 7.62 (d, 4H, *J* = 8.2 H_Z_), 7.49 (d, 4H), 7.44 (d, 2H, *J* = 8.2 H_Z_), 7.33 (d, 2H), 6.89 (d, 2H, *J* = 4.3 H_Z_), 6.72 (d, 2H), 2.47 (s, 3H, Ar–CH_3_), 0.28 (s, 18H, Si(CH_3_)_3_).

4,4’-difluoro-3,5-(4-(ethynyl)phenylethynyl)-8-(4-tolyl)-4-bora-3a,4a-diaza-*s*-indacene (M2): 100 μL tetrabutylammonium fluoride solution (1.0 mol L^−1^ in THF) was added into the solution of **1** (67 mg, 0.1 mmol) in THF (20 mL) at −78 °C under argon. After stirring for 15 min, the reaction mixture was poured into water (50 mL) and extracted with CH_2_Cl_2_ (3 × 50 mL). The organic layer was washed with water for twice and then dried over anhydrous Na_2_SO_4_ for 3 h. The solvent was evaporated under reduced pressure. The crude product was purified by silica gel column chromatography using petroleum ether/CH_2_Cl_2_ (1:1, *v/v*) as eluent to afford dark blue solid M2 (33 mg, 62.2% yield). FT-IR (KBr), *υ* (cm^−1^): 3294 (≡C–H), 2962, 2925, 2853 (CH_3_), 2202, 2157 (C≡C), 1610, 1568, 1537, 860 (Ar). ^1^H NMR (500 MHz, CDCl_3_) δ (ppm): 7.65 (d, 4H, *J* = 8.5 H_Z_), 7.52 (d, 4H), 7.44 (d, 2H, *J* = 8.0 H_Z_), 7.33 (d, 2H), 6.90 (d, 2H, *J* = 4.1 H_Z_), 6.73 (d, 2H), 3.24 (s, 2H, C≡CH), 2.47 (s, 3H, Ar–CH_3_). ^13^C NMR (125 MHz, CDCl_3_) δ (ppm): 142.7, 141.2, 137.4, 136.9, 132.2, 132.1, 131.0, 130.7, 130.5, 129.2, 123.8, 123.2, 122.8, 101.8, 85.0 (C≡C), 83.3 (HC≡C), 79.7 (HC≡), 29.7 (*C*H_3_). ESI–MS: *m/z* 511.3 [M-F]^−^. HRMS [M-F]^−^: calcd. for C_36_H_21_BFN_2_ 511.3747, found 511.3698.

4,4’-difluoro-2,6-(4-(trimethylsilylethynyl)phenylethynyl)-1,3,5,7-tetramethyl-8-(4-tolyl)-4-bora-3a,4a-diaza-*s*-indacene (2): M3 (590 mg, 1 mmol) and 1-ethynyl-4-(trimethylsilylethynyl)benzene (495 mg, 2.5 mmol) were dissolved in 150 mL of THF and 800 μL of Et_3_N under argon. Then PdCl_2_(PPh_3_)_2_ (35 mg, 0.05 mmol) and CuI (10 mg, 0.05 mmol) were added to this solution. After stirring at 60 °C for 10 h, the reaction mixture was poured into water (50 mL) and extracted with CH_2_Cl_2_ (3 × 50 mL). The organic layer was washed with water for twice and dried over anhydrous Na_2_SO_4_ for 3 h. The solvent was removed by evaporation under reduced pressure. The crude product was purified by silica gel column chromatography with petroleum ether/CH_2_Cl_2_ (1:1, *v/v*) as eluent to give orange solid 2 (465 mg, 63.7% yield). FT-IR (KBr), *υ* (cm^−1^): 2938, 2849 (CH_3_), 2214, 2135 (C≡C), 1629, 1566, 1390, 823 (Ar). ^1^H NMR (500 MHz, CDCl_3_) δ (ppm): 7.43 (d, 4H, *J* = 8.4 H_Z_), 7.39 (d, 4H), 7.36 (d, 2H, *J* = 8.0 H_Z_), 7.18 (d, 2H), 2.73 (s, 6H), 2.49 (s, 3H, Ar–CH_3_), 1.56 (s, 6H), 0.27 (s, 18H, Si(CH_3_)_3_).

4,4-difluoro-2,6-(4-(ethynyl)phenylethynyl)-1,3,5,7-tetramethyl-8-(4-tolyl)-4-bora-3a,4a-diaza-*s*-indacene (M4): 100 μL of tetrabutylammonium fluoride (1.0 mol L^−1^ in THF) was added into the solution of 2 (73 mg, 0.1 mmol) in THF (20 mL) at –78 °C under argon. After stirring for 15 min, the reaction mixture was poured into water (50 mL) and extracted with CH_2_Cl_2_ (3 × 50 mL). The organic layer was washed with water for twice and then dried over anhydrous Na_2_SO_4_ for 3 h. The solvent was removed by evaporation under reduced pressure. The crude product was purified by silica gel column chromatography with petroleum ether/CH_2_Cl_2_ (1:1, *v/v*) as eluent to give orange solid M4 (40 mg, 68.1% yield). FT-IR (KBr), *υ* (cm^−1^): 3312 (≡C–H), 2958, 2931, 2848 (CH_3_), 2199, 2101 (C≡C), 1623, 1549, 1516, 871 (Ar). ^1^H NMR (500 MHz, CDCl_3_) δ (ppm): 7.46 (d, 4H, *J* = 8.3 H_Z_), 7.42 (d, 4H), 7.36 (d, 2H, *J* = 7.9 H_Z_), 7.18 (d, 2H), 3.19 (s, 2H, C≡CH), 2.74 (s, 6H), 2.50 (s, 3H, Ar-CH_3_), 1.57 (s, 6H). ^13^C NMR (125 MHz, CDCl_3_) δ (ppm): 158.4, 147.1, 144.3, 143.2, 139.5, 132.1, 131.1, 130.1, 127.6, 124.5, 123.9, 121.7, 119.1, 96.0, 83.8 (C≡C), 83.3 (HC≡C), 78.9 (HC≡), 22.7, 21.5 (Ar–CH_3_), 13.5. ESI–MS: *m/z* 567.4 [M-F]^−^. HRMS [M-F]^−^: calcd. for C_40_H_29_BFN_2_ 567.2457, found 567.2446.

#### 2.3.2. Polymerization of Monomers

Synthesis of P2: A solution of M2 (51 mg, 0.1 mmol), 9,9-dioctyl-2,7-dibromofluorene (M2’) (55 mg, 0.1 mmol) and CuI (0.95 mg, 0.005 mmol) in dry THF (10 mL) and Et_3_N (1 mL) was degassed twice with nitrogen following the addition of Pd(PPh_3_)_4_ (3.56 mg, 0.005 mmol). After refluxing for 48 h under the protection of nitrogen, the reaction mixture was concentrated to 2 mL. Forty milliliters of methanol was added to the solution and stirred for 4 hours to precipitate the polymer. The precipitate was centrifuged and redissolved in THF (2 mL). Then the polymer was reprecipitated by dropwise adding the solution to 40 mL of methanol. The dissolution-reprecipitation operation was redone three times, and the resulted polymer was dried in vacuum oven at 45 °C for 24 hours as a dark blue solid (27.6 mg) in 31.1% yield. *M_w_* = 12.4 × 10^3^; *M_w_/M_n_* = 2.0 (GPC, polystyrene calibration). FT-IR (KBr), *υ* (cm^−1^): 2932, 2854 (CH_3_), 2199 (C≡C), 1547, 1252, 1159, 1034, 814 (Ar). ^1^H NMR (500 MHz, CDCl_3_) δ (ppm): 7.68–7.21 (br, Ar–H), 7.05–6.34 (br, H of BODIPY ring), 2.39 (br, CH_3_ linked at 8 position of BODIPY), 1.87 (br, CH_2_ linked at 8 position of Fluorene), 1.42–1.03 (br, CH_2_ of alkyl), 0.84 (br, CH_3_ of alkyl).

Synthesis of P1: P1 was synthesized from monomers M1 and M1’ according to the procedure for preparing P2 as a dark solid in 38.7% yield. *M_w_* = 10.7 × 10^3^; *M_w_/M_n_* = 1.9 (GPC, polystyrene calibration). FT-IR (KBr), *υ* (cm^−1^): 2927, 2860 (CH_3_), 2184 (C≡C), 1545, 1510, 1470, 1409, 803 (Ar). ^1^H NMR (500 MHz, CDCl_3_) δ (ppm): 7.88–7.23 (br, Ar–H), 6.85-6.54 (br, H of BODIPY ring), 2.48 (br, CH_3_ linked at 8 position of BODIPY), 2.08 (br, CH_2_ linked at 8 position of fluorene), 1.24–0.99 (br, CH_2_ of alkyl), 0.80 (br, CH_3_ of alkyl).

Synthesis of P3: P3 was synthesized from monomers M3 and M1’ according to the procedure for preparing P2 as a dark brown solid in 41.8% yield. *M_w_* = 11.8 × 10^3^; *M_w_/M_n_* = 1.8 (GPC, polystyrene calibration). FT-IR (KBr), *υ* (cm^−1^): 2924, 2853 (CH_3_), 2203 (C≡C), 1530, 1458, 1470, 1189, 821 (Ar). ^1^H NMR (500 MHz, CDCl_3_) *δ* (ppm): 7.76-7.12 (br, Ar–H), 2.77 (br, CH_3_ linked at 3, 5 position of BODIPY), 2.49 (br, CH_3_ linked at 8 position of BODIPY), 1.98 (br, CH_3_ linked at 1, 7 position of BODIPY), 1.28 (br, CH_2_ linked at 8 position of Fluorene), 1.20–0.98 (br, CH_2_ of alkyl), 0.77 (br, CH_3_ of alkyl).

Synthesis of P4: P4 was synthesized from monomers M4 and M2’ according to the procedure for preparing P2 as a dark solid in 33.8% yield. *M_w_* = 13.3 × 10^3^; *M_w_/M_n_* = 2.0 (GPC, polystyrene calibration). FT-IR (KBr), *υ* (cm^−1^): 2921, 2846 (CH_3_), 2196 (C≡C), 1539, 1375, 1108, 818 (Ar). ^1^H NMR (500 MHz, CDCl_3_) δ (ppm): 7.54–7.16 (br, Ar–H), 2.69 (br, CH_3_ linked at 3, 5 position of BODIPY), 2.49 (br, CH_3_ linked at 8 position of BODIPY), 1.98 (br, CH_3_ linked at 1, 7 position of BODIPY), 1.55 (br, CH_2_ linked at 8 position of fluorene), 1.29–1.07 (br, CH_2_ of alkyl), 0.98 (br, CH_3_ of alkyl).

## 3. Results and Discussion

### 3.1. Synthesis and Structural Characterization

**Scheme 2:**
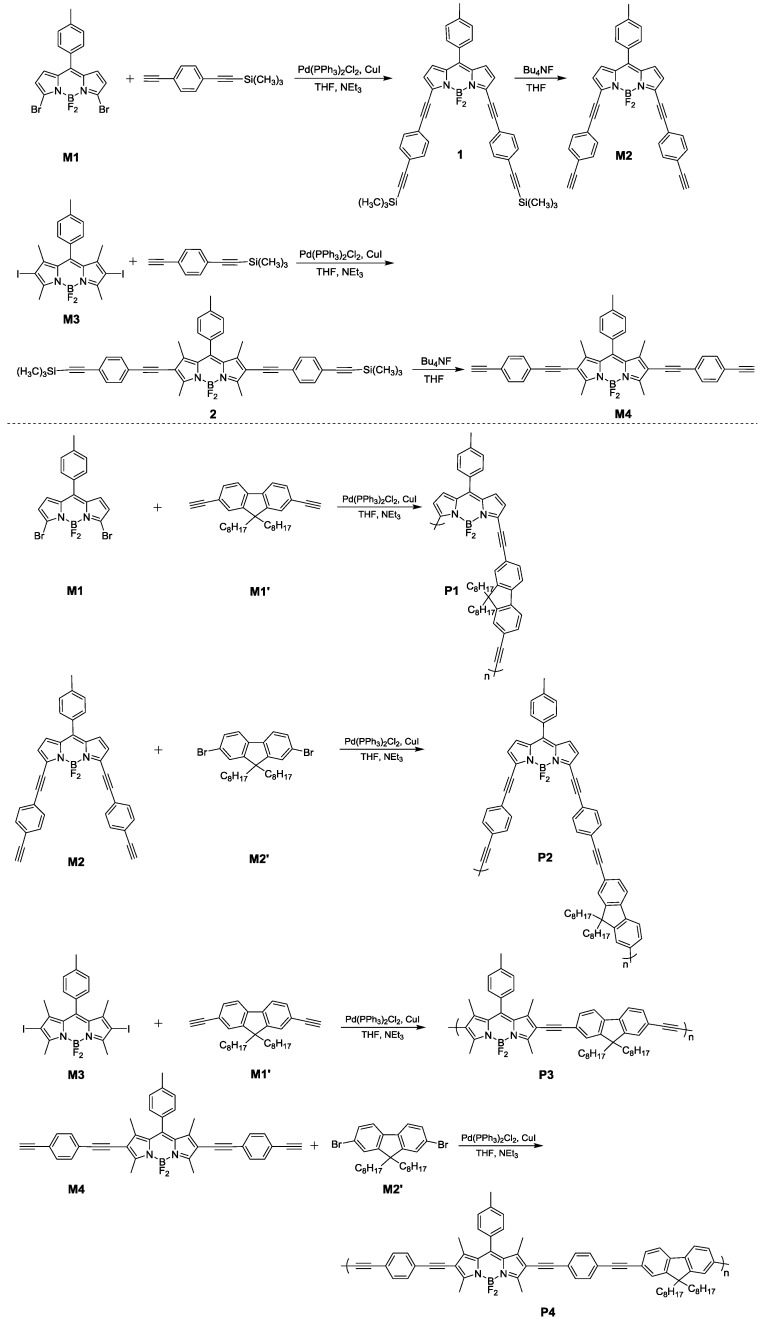
Synthetic routes for monomers (M2 and M4) and polymers (P1, P2, P3 and P4).

The synthetic routes to the BODIPY monomers and polymers are shown in Scheme 2. M1 and M3 were synthesized according to the literature. As shown in Scheme 2, monomers M2 and M4 were synthesized from M1 and M3 using the same method, respectively. In the case of M2, the trimethylsilyl protected compound 1 was first conveniently synthesized in 50.4% yield using the Sonogashira coupling of M1 with 1-ethynyl-4-(trimethylsilylethynyl)benzene in the presence of a catalytic amount of Pd(PPh_3_)_2_Cl_2_ and CuI with Et_3_N. After desilylation with tetrabutylammonium fluoride in THF, M2 was obtained in good yield (62.2%). The chemical structures of the final monomers were characterized by ^1^H NMR, ^13^C NMR, FT-IR, ESI–MS and HRMS spectrometry as shown in the Electronic Supplementary Information (Figures S1–S18).

The polymerization was then accomplished by Sonogashira coupling reaction of BODIPY derivative monomers with substituted fluorene monomers in ~40% yield (Scheme 2). All the polymers can dissolve in common organic solvents, such as THF, CH_2_Cl_2_, CHCl_3_ and toluene. The weight-average molecular weight (*M_w_*) and polydispersity index (PDI) of the polymers measured by GPC were 10.7 × 10^3^ and 1.9 for P1, 12.4 × 10^3^ and 2.0 for P2, 11.8 × 10^3^ and 1.8 for P3, and 13.3 × 10^3^ and 2.0 for P4, respectively.

**Figure 1:**
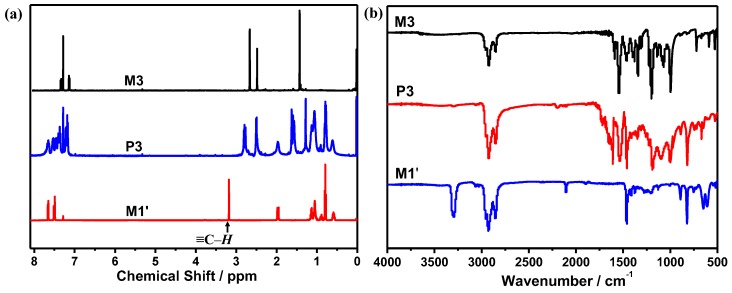
^1^H NMR spectra (**a**); and FT-IR spectra (**b**) of monomers (M1’ and M3) and polymer P3.

The structures of the polymers were characterized by ^1^H NMR and FT-IR spectroscopy (Figure 1). In the case of P3, as depicted in the ^1^H NMR spectra, a characteristic absorption singlet peak at *δ* 3.19 ppm assigned to the acetylene proton of M1’ completely disappeared in the spectrum of P3. Besides, the resonance peaks of P3 were broader than those of the monomers M3 and M1’, due in part to their longer rotational correlation times. These results indicate that the acetyl triple bonds of M3’ have been completely involved in the polymerization. The FT-IR spectra of monomers and P3 are shown in Figure 1b. As shown in Figure 1b, monomer M1’ displayed the characteristic ≡C–H stretching vibrations at 3300 cm^−1^. After polymerization, the characteristic ≡C–H stretching vibration completely disappeared, and the characteristic C≡C stretching vibrations at 2110 cm^−1^ was shifted to 2203 cm^−1^ in the spectrum of P3, which further proved the efficient coupling reaction and the successful preparation of the target polymer. The same results can be obtained by comparing the ^1^H NMR and FT-IR spectra of other monomers with their corresponding polymers presented in the ESI.

### 3.2. Thermal Properties

**Figure 2:**
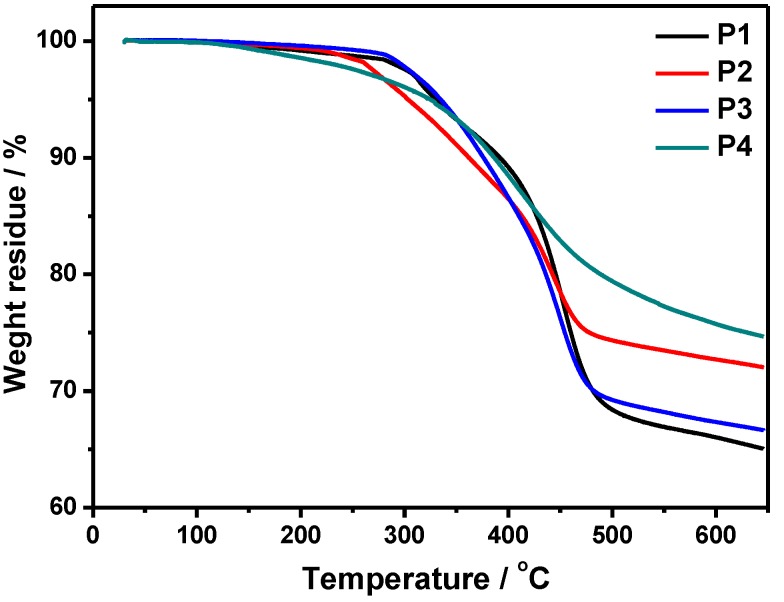
TGA curves of the polymers (P1, P2, P3 and P4).

The thermal stability of these polymers were investigated by thermal gravimetric analyzer (TGA) at the heating rate of 10 °C min^−1^ under N_2_ atmosphere. As shown in Figure 2, the decomposition temperature (*T*_d_) of 5% weight loss for P1, P2, P3 and P4 were 329, 303, 334 and 324 °C, respectively, which indicated that the obtained polymers have good thermal stability, and could provide a desirable thermal property for opto-electronic materials. Besides, it could be found that P3 and P4 showed better thermal stability than P1 and P2, respectively, which may be due to the existence of more effective electron delocalization in the polymerization at the 3,5 positions than that at the 2,6 positions. Moreover, the incorporation of *p*-diacetylenebenzene into the polymer main chain led to poorer thermal stability of the polymer by comparing P1 with P2 or P3 with P4.

### 3.3. Optical Properties

**Figure 3:**
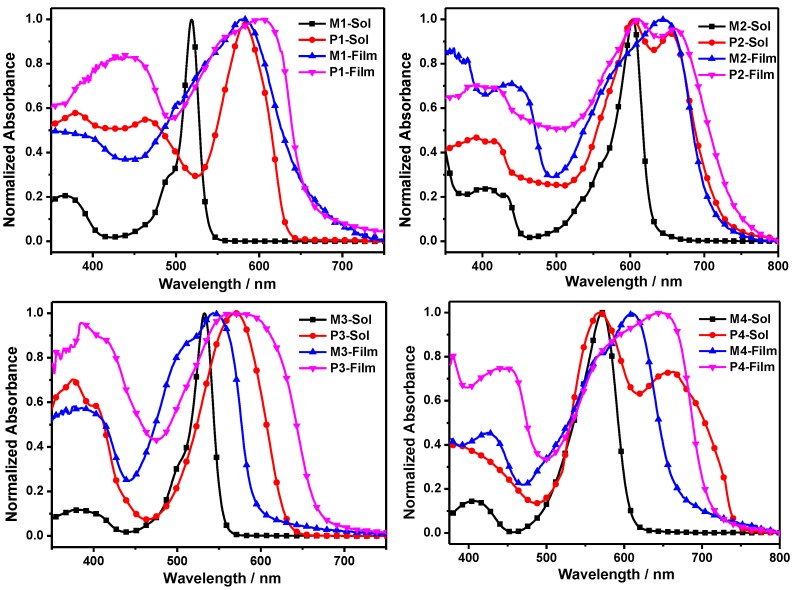
Normalized absorption spectra of monomers (M1, M2, M3 and M4) and copolymers (P1, P2, P3 and P4) in THF solution and films.

The normalized absorption spectra of all the BODIPY monomers and polymers in dilute THF solution and thin films are shown in Figure 3, and the optical data are summarized in Table 1. From Figure 3, in THF solution, M1 showed a strong S_0_–S_1_ (π–π*) transition with a maximum at 519 nm and a weak broad band around 365 nm ascribed to the S_0_–S_2_ (π–π*) transition, characteristic of the typical absorption spectrum of a BODIPY chromophore. When attached to the 3,5 positions of BODIPY with two *p-*diacetylenebenzene units, the absorption peaks of M2 were red-shifted by 84 nm compared with that of M1, due to the increased π-conjugation. Similarly, the absorption maxima of M4 exhibited a bathochromic shift of 39 nm in comparison with that of M3. After polymerization, the absorption peak of P1 red-shifted to 550–610 nm, and the absorbance of P1 at 350–500 nm was apparently larger than that of M1. Similarly, compared to the absorption spectra of their respective BODIPY monomers, other copolymers also showed broad absorption in the wavelength range from 300 to 750 nm with red-shifted and broader absorption peaks, due to the significant extended conjugation length of polymer backbone and the intramolecular charge transfer (ICT) from fluorene to BODIPY. In addition, the absorption spectra of both P2 and P4 possessed a vibronic shoulder peak at ~660 nm, which imply the existence of ordered aggregation and strong π–π stacking in solution [31].

In solid films, the absorption spectra of all the monomers and polymers exhibited red shifts and broader peaks relative to their corresponding solution absorption, indicative of strong interactions. For example, the spectrum of M1 was broadened and red-shifted with a peak at 580 nm. After polymerization, the polymers presented broader and red-shifted absorption peaks in contrast to the BODIPY monomers. In addition, in Table 1, P2 and P4 exhibited bathochromic shifts in comparison with P1 and P3, respectively, because of longer conjugation by the ethynylphenyl group. Moreover, the absorption maxima of P1 and P2 exhibited bathochromic shifts compared to those of P3 and P4, respectively, which suggested that substitutions at 3,5-positions of BODIPY provided more effective π-conjugation than substitutions at 2,6 positions.

**Figure 4:**
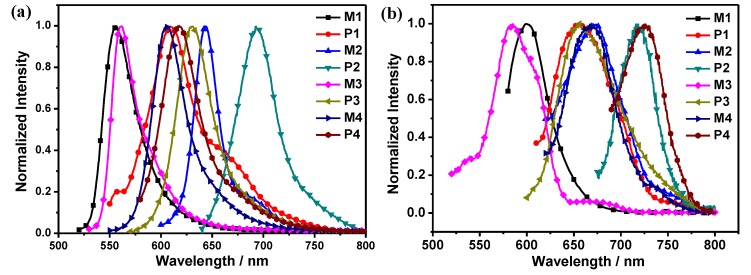
Normalized fluorescence emission spectra of monomers (M1, M2, M3 and M4) and copolymers (P1, P2, P3 and P4) in: THF (**a**); and films (**b**).

The emission spectra of monomers and copolymers in THF solution and films are shown in Figure 4 and ESI. In Figure 4, the change trends were the same as those of their absorption spectra and extended π-conjugation length of the copolymers results in significant bathochromic shifts of fluorescence maxima and broader emission peaks compared to their corresponding starting BODIPY monomers (Table 1). In solution, M1 and M3 displayed the maximum emission peaks at ~560 nm, while M2 and M4 blue-shifted to 644 and 607 nm, respectively. After polymerization, the fluorescent spectrum of P1 was broadened and red-shifted with peak at 609 nm. Similarly, the fluorescence spectra of P2, P3 and P4 also showed broadened peaks and red shifts in comparison with those of their corresponding monomers. Besides, from Table 1, it can be seen that P1, P2 and P4 exhibited lower fluorescence quantum yields in comparison with those of BODIPY monomers originated from the ICT excited states, and P3 showed the highest quantum yield with a value of 18.7% among all the polymers. Monomer M3 exhibits very low fluorescence quantum yield compared to other monomers because of the heavy atom effect of iodine substituents.

The fluorescence properties of the monomers and polymers in the solid state were then measured (Figure 4b). Compared with those in solution, the emission spectra of the monomers in thin films preserved most of the spectra features and displayed obvious red shifts due to the strong π–π stacking. After polymerization, the fluorescence spectra of the polymers in the thin films were broadened and redshifted because of the extension of conjugation and strong intermolecular interaction.

### 3.4. Electrochemical Properties 

The electrochemical properties of the copolymers were studied by cyclic voltammetry (CV). The potentials were internally calibrated using the ferrocene/ferrocenium (Fc/Fc^+^) of the redox couple (4.8 eV below the vacuum level). The HOMO and LUMO energy levels were calculated from the onset oxidation and reduction potential of the redox curves. The CV curves are shown in Figure S19 and the electrochemical data are listed in Table S1. In Table S1, the HOMO and LUMO energy levels of the polymers are −5.22 and −3.36 eV for P1, −5.08 and −3.41 eV for P2, −5.14 and −3.25 eV for P3, and −5.11 and −3.35 eV for P4, respectively. P2 and P4 show higher HOMO energy levels relative to P1 and P3, respectively, due to the extended conjugation length and thus stronger electron-donating ability. P1 and P2 show lower LUMO energy levels compared to P3 and P4, respectively. The band gaps for P1, P2, P3 and P4 are 1.89, 1.67, 1.86 and 1.75 eV, respectively.

**Figure 5:**
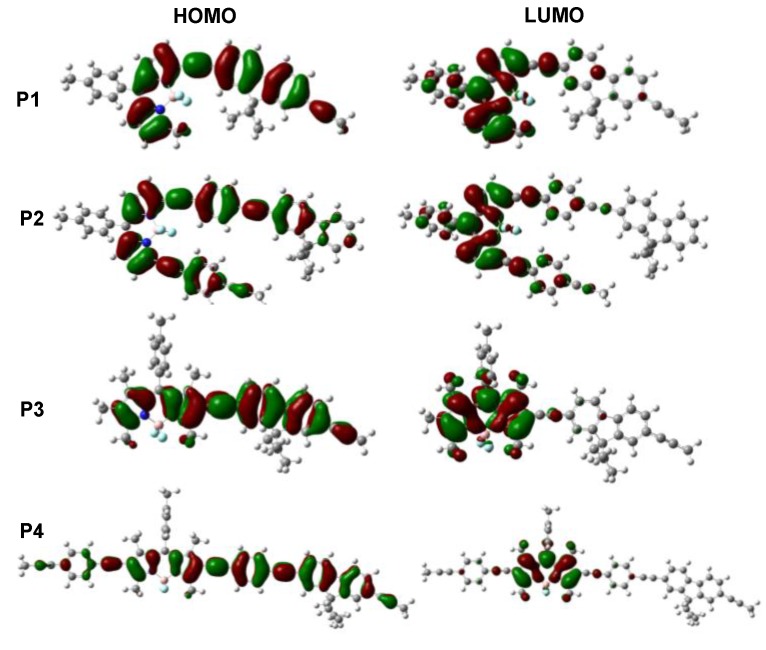
Frontier molecular orbitals of polymers using DFT evaluated at the B3LYP/6-31G(d) level.

Density functional theory (DFT) calculations were further performed on the corresponding repeating units of the four polymers to gain a better understanding of the electrochemical properties. The frontier molecular orbital energy levels and the corresponding HOMO and LUMO surface plots are shown in Figure 5 and Table S1. The variation trends for molecular orbital energy levels are in accordance with the results obtained from the CV measurements. In Figure 5, the HOMO surfaces are well spread over the whole of the conjugated backbones. Compared with P1 and P3, longer HOMO delocalization length for P2 and P4 would contribute to higher HOMO energy levels. The electron density of the LUMO for P1 and P2 is mainly localized on the BODIPY and adjacent acetylene and benzene, while the LUMO of P3 and P4 is mainly localized on the BODIPY and acetylene units. Compared with P3 and P4, the longer LUMO delocalization length for P1 and P2 would result in lower LUMO energy levels. These results are in concert with the experimental results. The theoretical calculation results indicate that changing the connectivity and conjugation length has great effect on the optical and electrical characteristics.

### 3.5. Sensing Properties

**Figure 6:**
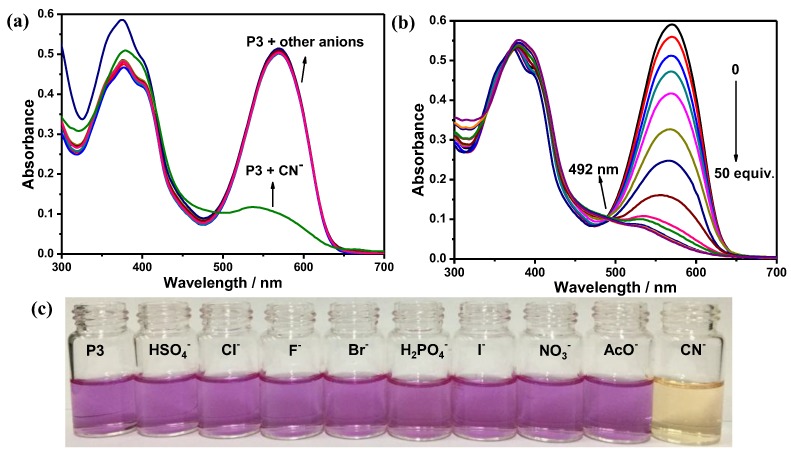
(**a**) UV-Vis spectra of P3 ([RU] = 30 μM) in THF/H_2_O (98:2, *v/v*) in the presence of 20 equiv. of different anions; (**b**) UV-Vis spectral changes of P3 ([RU] = 30 µM) in THF/H_2_O (98:2, *v/v*) upon addition of an increasing concentration of CN^−^ (0, 120, 240, 270, 300, 330, 360, 390, 420, 450, 480, 510, 540, 570, 600, 700, 800, 900, 1000, 1200 and 1500 µM); and (**c**) visual images observed for P3 solutions upon addition of 50 equiv. each anion.

These polymers were evaluated for potential responses to anions. The selectivity of polymers with F^−^, Cl^−^, Br^−^, I^−^, CN^−^, AcO^−^, HSO_4_^−^, H_2_PO_4_^−^, NO_3_^−^ using tetrabutyl-ammonium salts in THF/H_2_0 (98:2, *v/v*) were investigated by UV-Vis and fluorescence spectroscopy, and naked eye colour changes. Upon the addition of 20 equiv. of various anions to all the four polymers in THF/H_2_O (98:2, *v/v*) solutions ([RU] = 30 μM), evident changes of the absorption spectra induced by CN^−^ could be observed (Figure 6). As shown in Figure 6a, in the case of P3, upon the addition of CN^−^, the initial absorption bands at 569 nm were blue-shifted to 539 nm along with the obvious decrease of the absorbance. However, any changes could hardly be found in presence of other anions. From Figure 6b, with the addition of an increasing concentration of CN^−^, gradual reduction of the absorbance and blue shift of the absorption band at 569 nm were observed, and clear isosbestic points at 492 nm was found, indicative of the formation of new species [6,32,33]. More importantly, the changes of the solution color from purple to faint yellow can be clearly observed by the naked eyes upon addition of 20 equiv. of CN^−^ ions to P3 (Figure 6c), while the other anions hardly induced any significant color change, which indicated that naked-eye selective detection of CN^−^ became possible.

**Figure 7:**
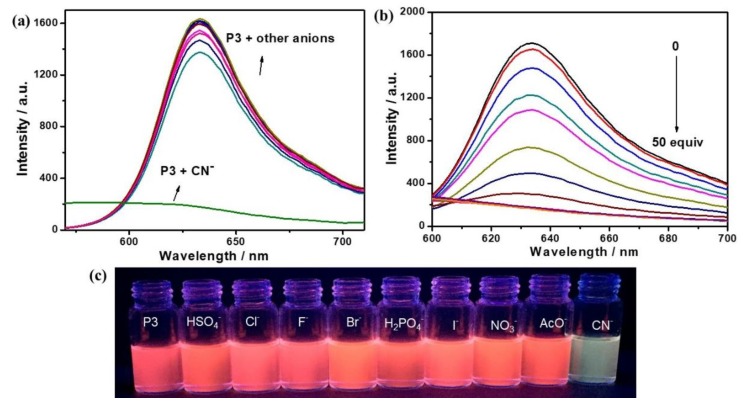
(**a**) Fluorescence spectra of P3 ([RU] = 30 µM) in THF/H_2_O (98:2, *v/v*) in the presence of 20 equiv. of different anions, λ_ex_ = 365 nm; (**b**) fluorescence spectral changes of P3 ([RU] = 30 µM) in THF/H_2_O (98:2, *v/v*) upon addition of an increasing concentration of CN^−^ (0, 120, 240, 270, 300, 330, 360, 390, 420, 450, 480, 510, 540, 570, 600, 700, 800, 900, 1000, 1200 and 1500 µM), λ_ex_ = 365 nm; and (**c**) fluorescence observed for P3 solutions upon addition of 50 equiv. each anion under the excitation of 365 nm.

The sensing behavior was further studied by fluorescence spectra. In the case of P3, as shown in Figure 7a, the fluorescence intensity of P3 at 633 nm was significantly quenched in the presence of cyanide anions, while it gave no distinct response to other anions. Titration of CN^−^ to the solution of P3 (Figure 7b), gradual decrease of fluorescence intensity at 633 nm was observed with the molar ratio increase of CN^−^ from 1:0 to 1:50 equiv. Indeed, besides the visible color change discussed above, the addition of CN^−^ also led to a significant quenching of the strong pink fluorescence as shown in Figure 7c. Other polymers showed similar obvious changes in the UV-Vis absorption and fluorescence spectra upon addition of CN^−^ ions, which were shown in Figures S20–S28. Based on the fluorescent titration data, the detection limit of P3 was estimated to be 33.2 µM according to the definition of IUPAC (3s/k, three times blank standard deviation/slope of analytical curve, Figure S29). Similarly, the detection limit of P1, P2 and P4 were 165.7, 89.4 and 22.4 µM, respectively. These results demonstrated these polymers could be used as selective and sensitive colorimetric probe for cyanide anions.

**Figure 8:**
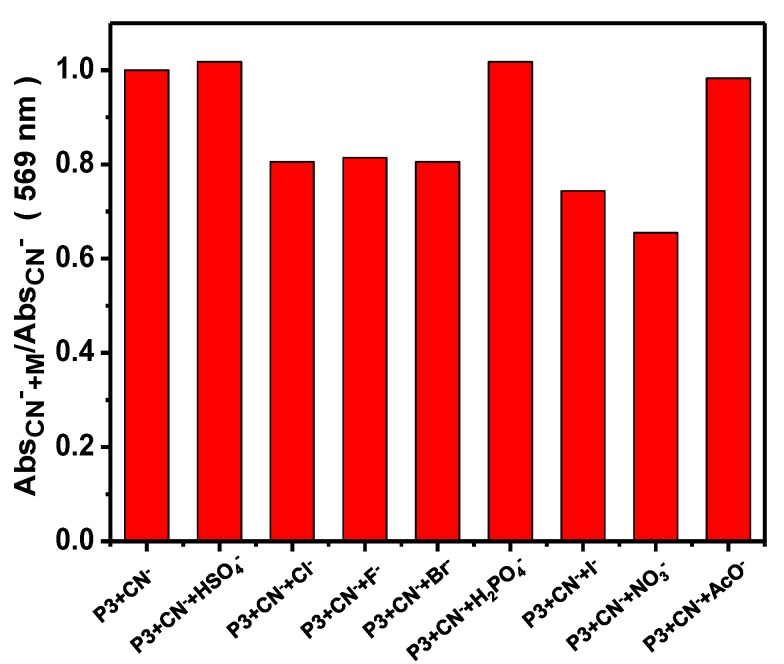
Changes in the UV-Vis absorbance at 569 nm of P3 ([RU] = 30 µM) containing 20 equiv. of CN^-^ anions upon the addition of 20 equiv. of other competing anions in THF/H_2_O (98:2, *v/v*).

To further demonstrate the preferential selective detection of CN^−^, competition experiments were further carried out by addition of 20 equiv. of other tested anions to P3 solution in the presence of 20 equiv. of CN^−^ anion and the results are shown in Figure 8. From Figure 8, the absorbance of P3 at 569 nm was hardly interfered by HSO_4_^−^, H_2_PO_4_^−^ and AcO_4_^−^, and was changed by 20%–35% by Cl^−^, F^−^, Br^−^, I^−^ and NO_3_^−^. The results suggest that the CN^−^ induced UV-Vis absorption response of P3 solution can coexist in other common anions. Other polymers (P1, P2 and P4) exhibited the similar phenomenon, indicative of the exclusive sensitivity of polymers towards CN^−^ (Figures S22, S25 and S28).

To gain further insight into the nature of polymers-cyanide interactions, the changes of ^1^H NMR spectra produced via the addition of cyanide anion to the M-BODIPY and P3 solutions were monitored. As depicted in Figure S30, the two doublet peaks at 7.24 and 7.11 ppm assigned to the phenyl protons at the 8 position of BODIPY unit were shifted to 7.12 and 6.82 ppm upon the addition of CN^−^ to a THF-*d*_8_ solution of M-BODIPY, respectively. These observations are consistent with the previous reported results [6], indicating that the BODIPY core may be broken after the addition of CN^−^ and new species were formed. The ^1^H NMR spectra of P3 before and after the addition of CN^−^ showed the similar results (Figure S30). The two peaks at 7.71 and 7.54 ppm assigned to the phenyl protons at the 8 position of BODIPY unit were similarly shifted to 7.55 and 7.47 ppm, respectively. Besides, the M-BODIPY exhibited similar UV-Vis and fluorescence spectral changes upon the addition of CN^−^ (as shown in Figure S31). Thus, the selective response of P3 to CN^−^ is by the decomposition of the BODIPY core.

## 4. Conclusions

In this work, four novel BODIPY-containing conjugated polymers with different connectivity on BODIPY and conjugation length were successfully synthesized. All copolymers show broad absorption in the wavelength region from 300 to 750 nm and display significant red shifts of both their absorption and fluorescence spectra maxima compared with their initial BODIPY monomers due to the significant extension of π-conjugation. The absorption and fluorescence spectra peaks of P2 and P4 with fluorene and BODIPY connected by *p*-diacetylenebenzene exhibited obvious red shifts compared with those of P1 and P3, respectively, because of their longer conjugation length. Besides, the red-shifted absorption and fluorescence peaks and longer LUMO delocalization length for P1 and P2 relative to those for P3 and P4, respectively, demonstrate that polymers with connectivity at 3,5-positions of BODIPY possess more effective conjugation than those at 2,6-positions. Moreover, the BODIPY-based copolymers exhibited selectively sensitive responses to cyanide anions, resulting in great blue-shift and decreased absorbance of UV-Vis absorption spectra, and significant quenching of the fluorescence intensity while they hardly displayed any responses to other ions, such as F^−^, Cl^−^, Br^−^, I^−^, AcO^−^, HSO_4_^−^, H_2_PO_4_^−^, and NO_3_^−^. The results demonstrated that BODIPY-based conjugated copolymers are highly promising for chemical and biological sensing applications.

## Figures and Tables

**Table 1:** Optical data of the monomers and copolymers.

Compound	λ_max, sol_ (nm)	λ_max, film_ (nm)	λ_em, sol_ (nm)	Φ_f_ (%)	λ_em, film_ (nm)
M1	519	580	557	30.4	600
P1	582	554, 605	609	1.9	655
M2	603	646	644	47.5	673
P2	604, 654	612, 662	693	10.6	718
M3	533	544	562	1.6	584
P3	569	575	633	18.7	656
M4	572	610	607	37.9	669
P4	568, 664	648	618	3.1	725

## Supplementary Materials

The supplementary materials are available online at www.mdpi.com/2073-4360/9/10/512/s1.
