# Supplementary material for: Conjugated Polymers Containing BODIPY and Fluorene Units for Sensitive Detection of CN− Ions: Site-Selective Synthesis, Photo-Physical and Electrochemical Properties
Source: Polymers (Basel). 2017 Oct 14;9(10):512. doi: 10.3390/polym9100512 (PMC6418624; doi:10.3390/polym9100512)
Supplement: Supplementary file 1 [file polymers-09-00512-s001.pdf]

polymers MDPI

Supplementary Materials: Conjugated Polymers Containing BODIPY and Fluorene Units for Sensitive Detection of CN^- Ions: Site-Selective Synthesis, Photo-Physical and Electrochemical Properties

Tian He,[+] Danting Tang,[+] Cuiling Lin, Xi Shen, Chenjie Lu, Luonan Xu, Zhengye Gu, Zheng Xu, Huayu Qiu, Qian Zhang* and Shouchun Yin*

Table of Contents

1. ^{1}H, ^{13}C NMR and FTIR spectra of monomers···P2-7
2. ^{1}H NMR and FTIR spectra of polymers···P7-10
3. Cyclic voltammograms of the polymers···P11
4. Experimental and calculated electrochemical data of polymers···P11
5. Spectroscopic responses of polymers to anions···P12-16
6. Plot of fluorescence intensity changes of polymers versus various concentrations of CN^- anions···P16
7. Partial ^{1}H NMR spectra of BODIPY and **P3** before and after the addition of 20 equiv. $[(n\text{-}Bu)_4N]^+CN^-$···P17
8. Spectroscopic responses of BODIPY monomers to anions ···P18

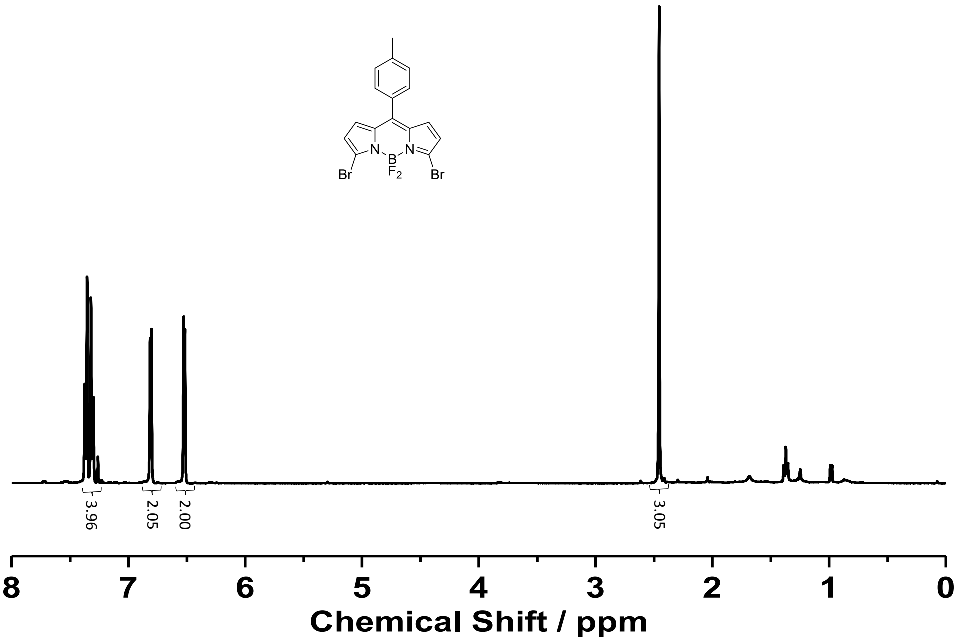

Figure S1. ¹H NMR spectrum of M1.

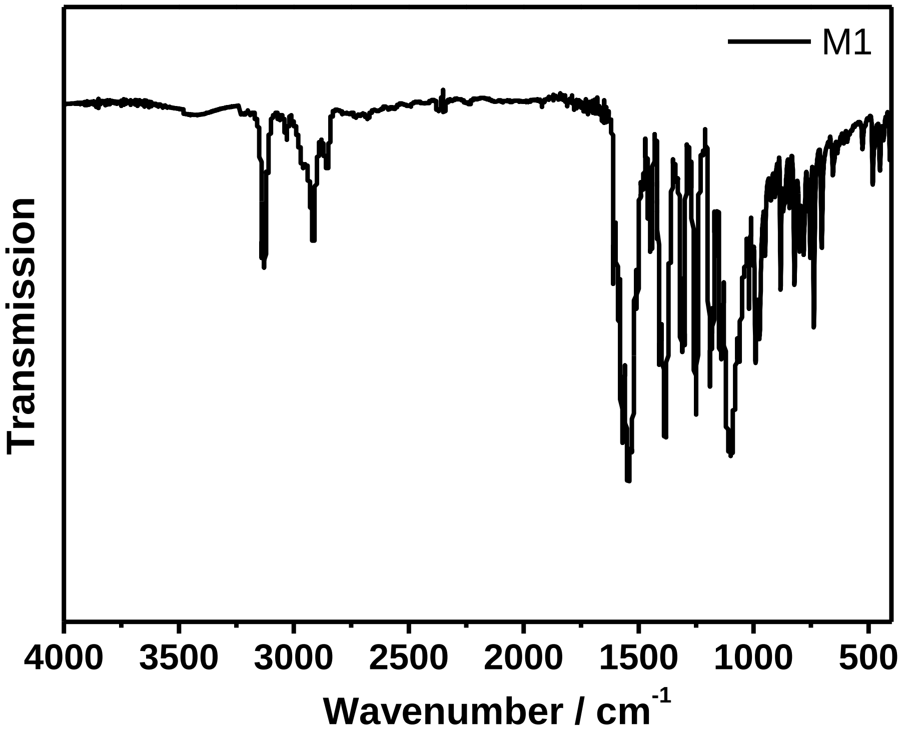

Figure S2. FTIR spectrum of M1.

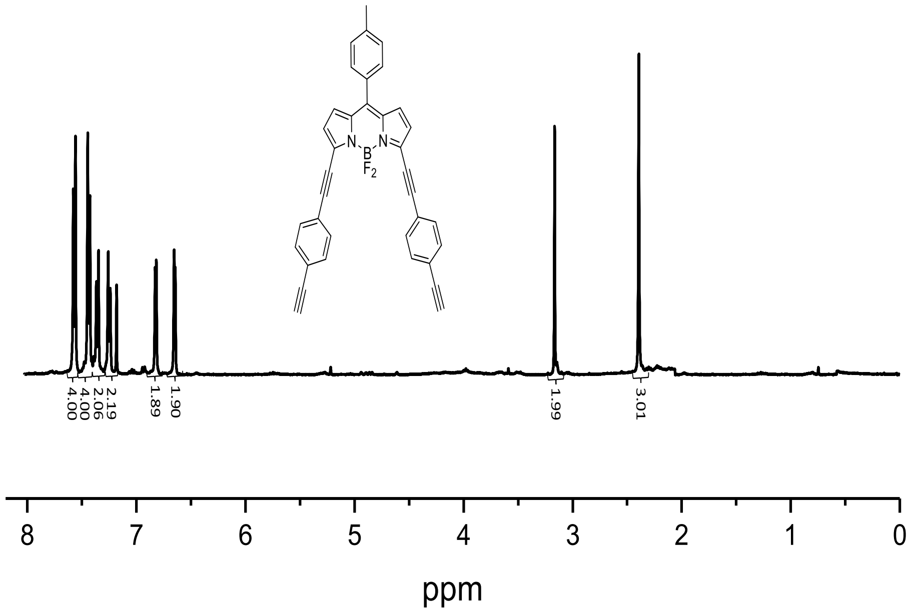

Figure S3. ^{1}H NMR spectrum of M2.

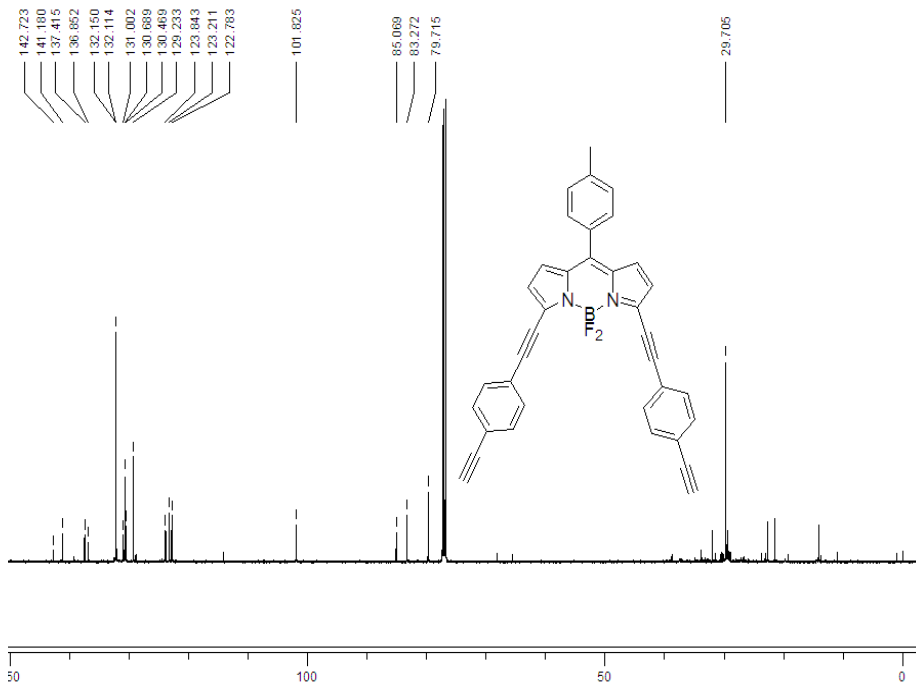

Figure S4. ^{13}C NMR spectrum of M2.

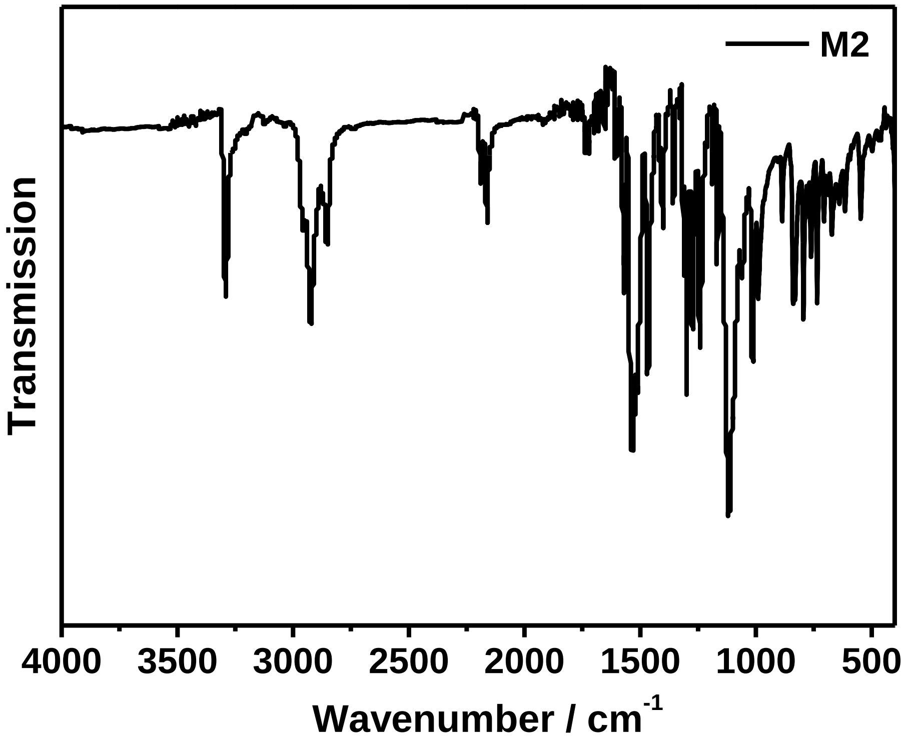

Figure S5. FTIR spectrum of M2.

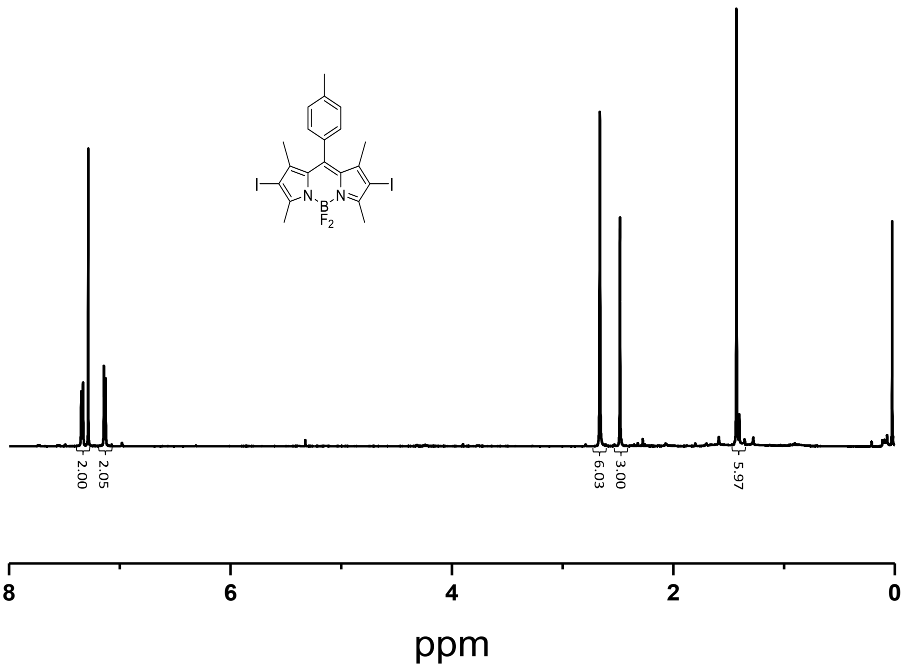

Figure S6. ^{1}H NMR spectrum of M3.

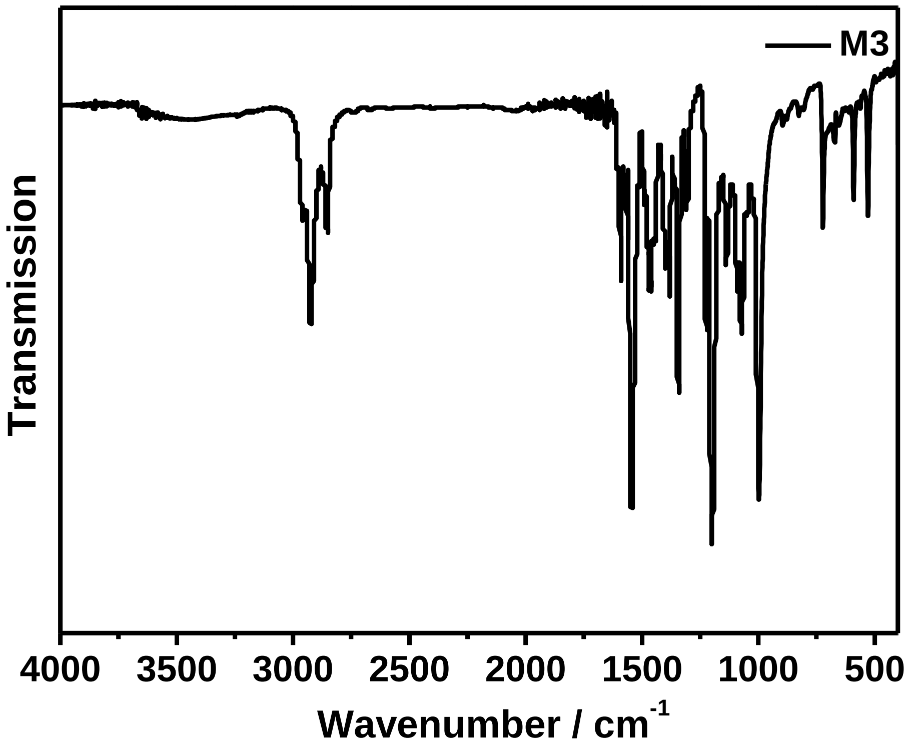

Figure S7. FTIR spectrum of M3.

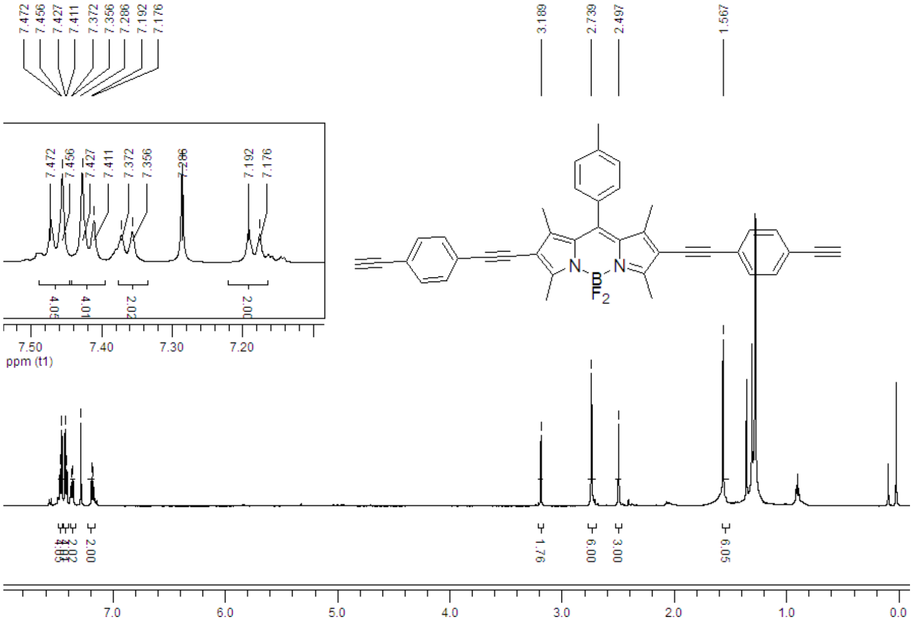

Figure S8. ^{1}H NMR spectrum of M4.

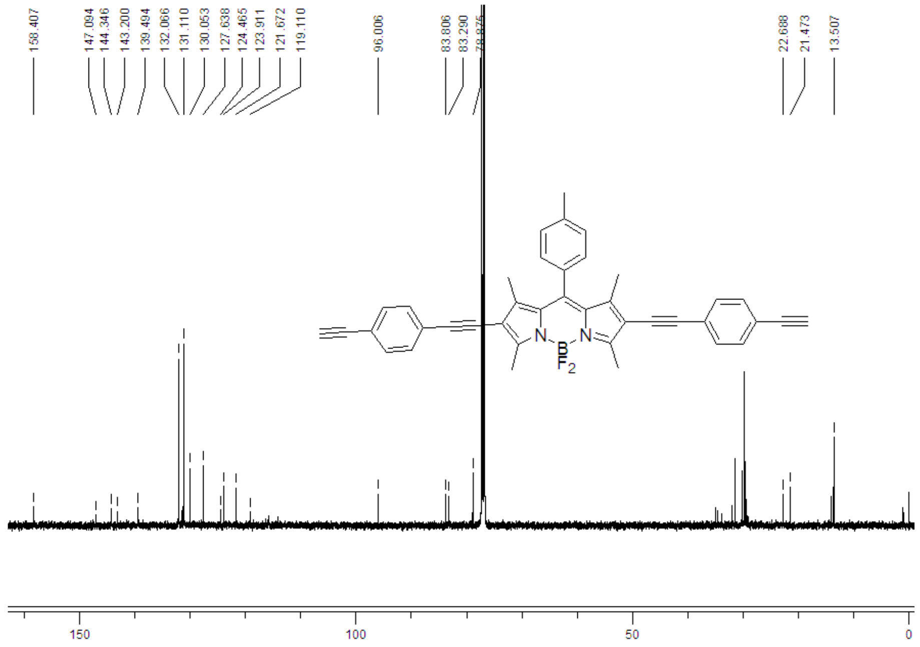

Figure S9. ^{13}C NMR spectrum of M4.

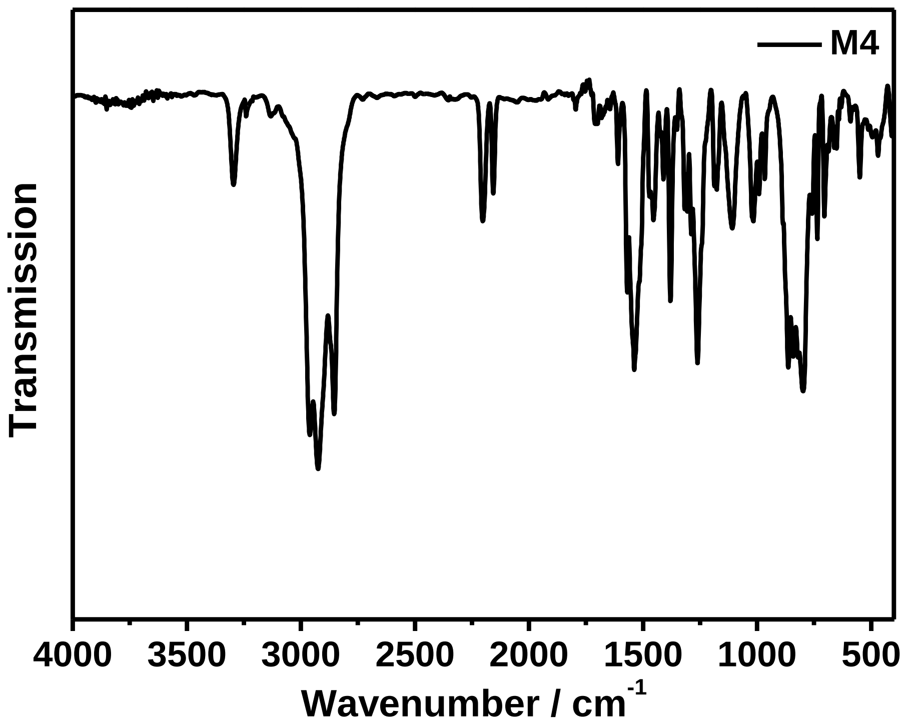

Figure 10. FTIR spectrum of M4.

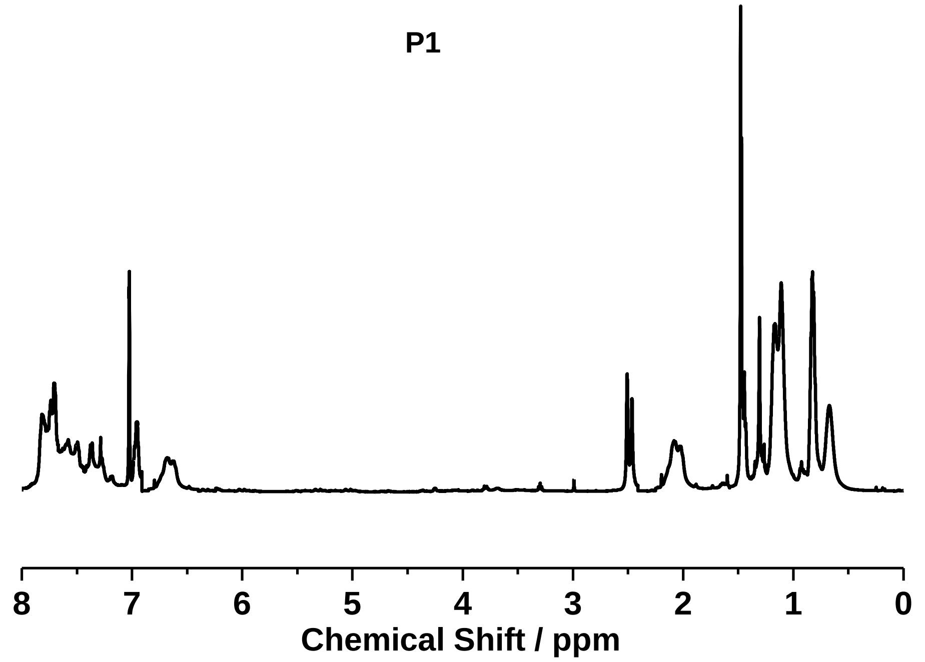

Figure S11. ^1H spectrum of P1.

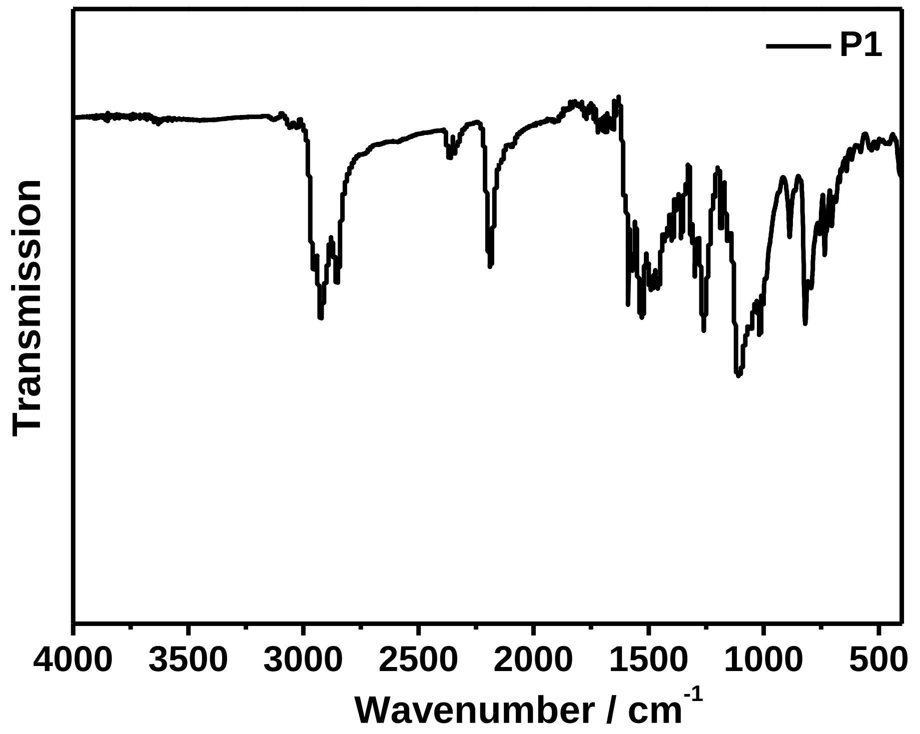

Figure S12. FTIR spectrum of P1.

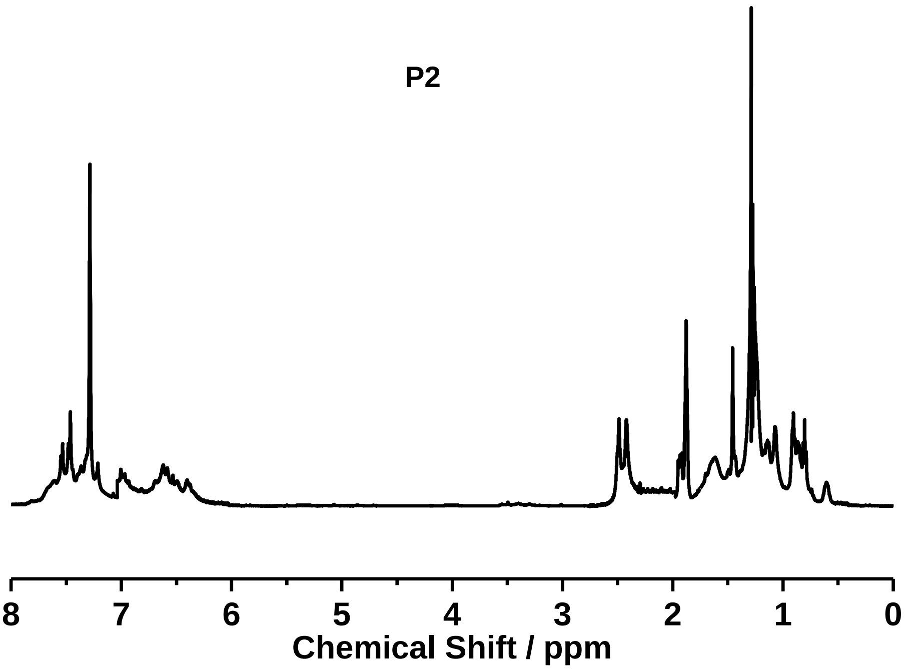

Figure S13. ^{1}H spectrum of P2.

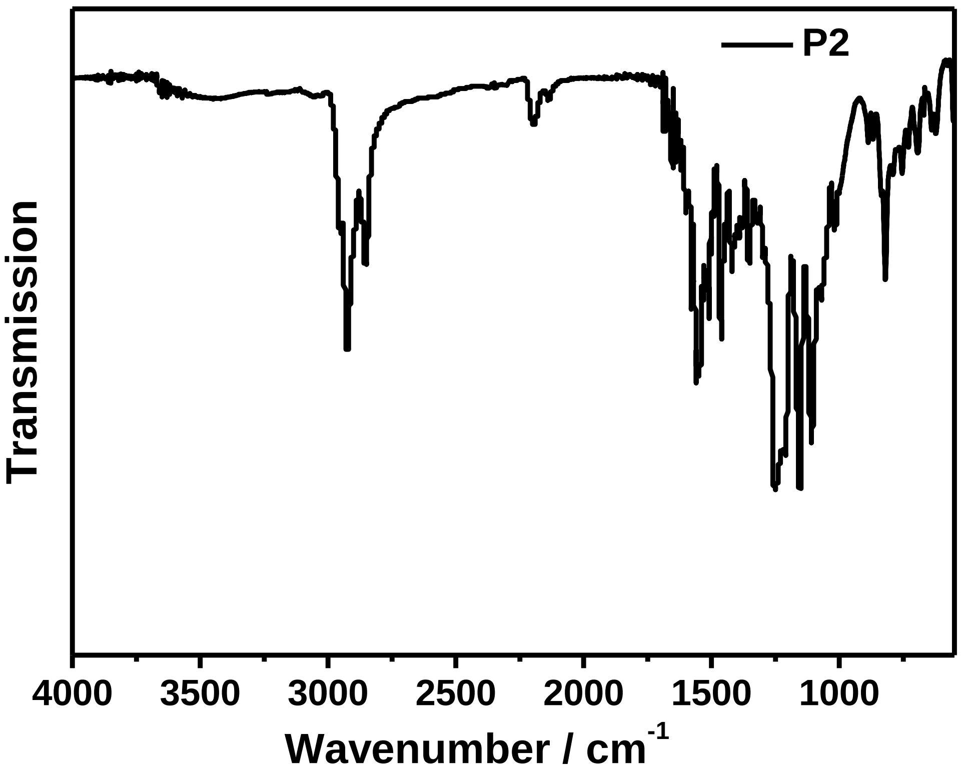

Figure S14. FTIR spectrum of P2.

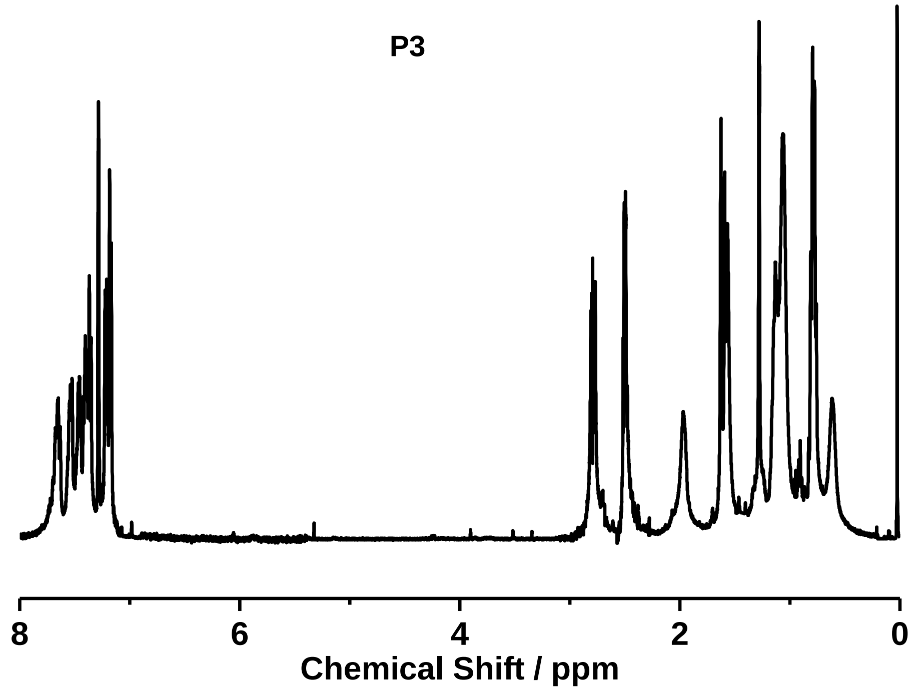

Figure S15. ^{1}H spectrum of P3.

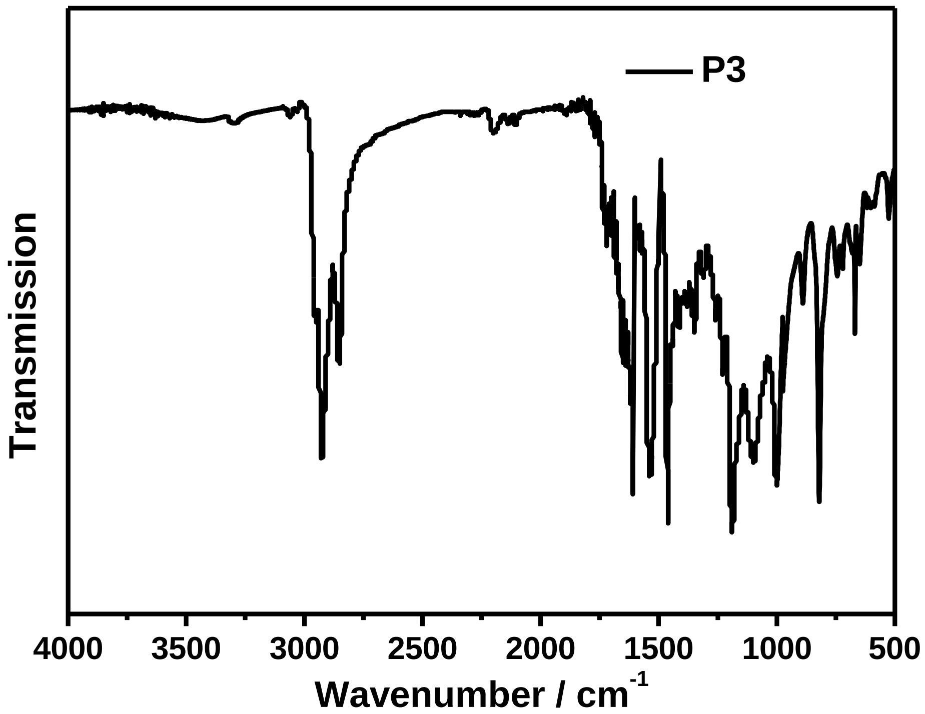

Figure S16. FTIR spectrum of P3.

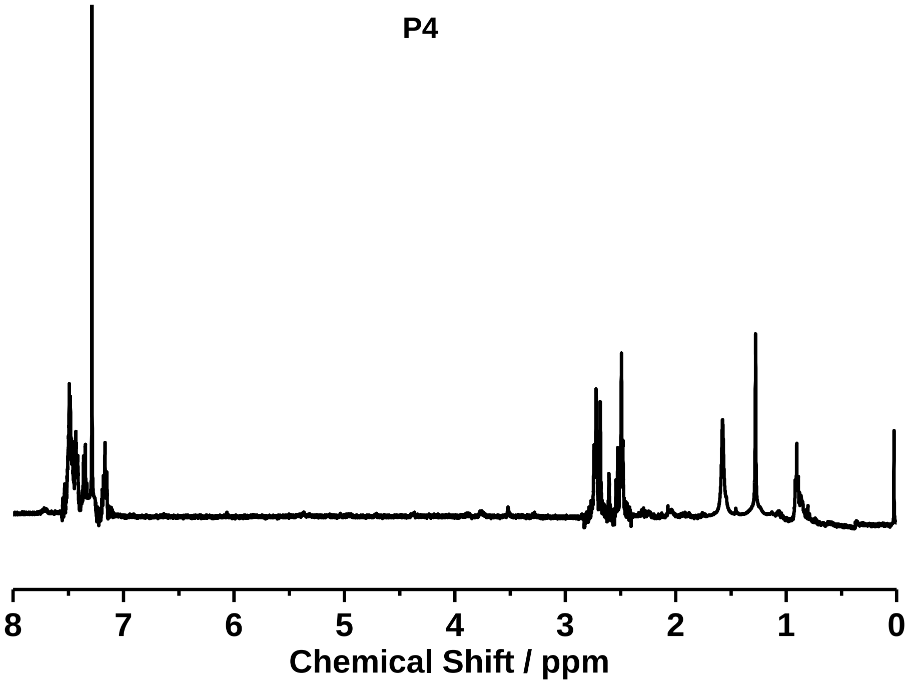

Figure S17. ^{1}H spectrum of P4.

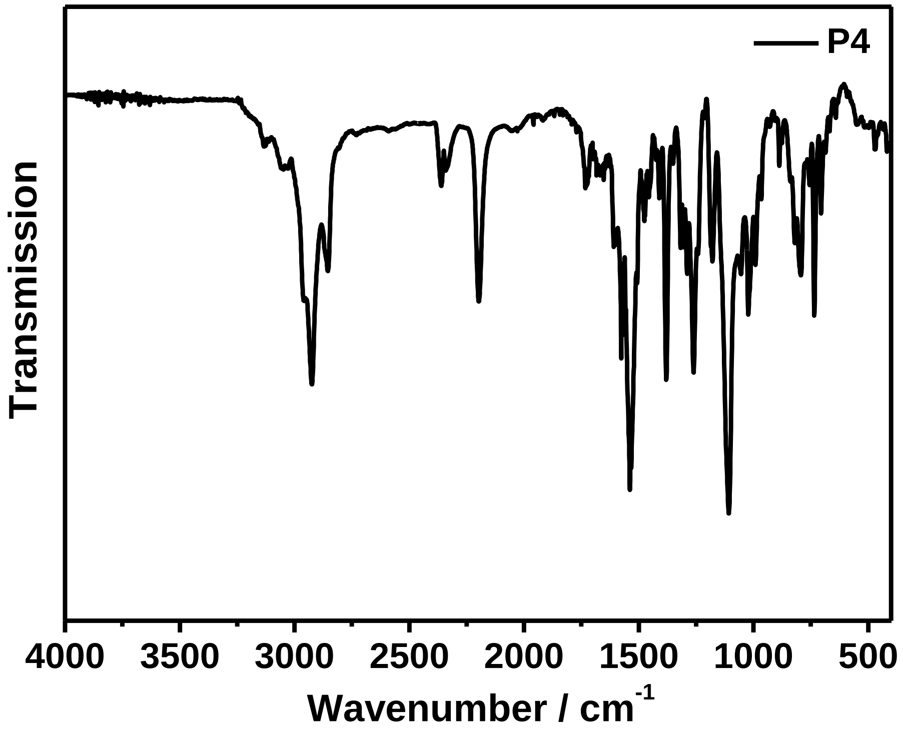

polymers MDPI

Figure S18. FTIR spectrum of P4.

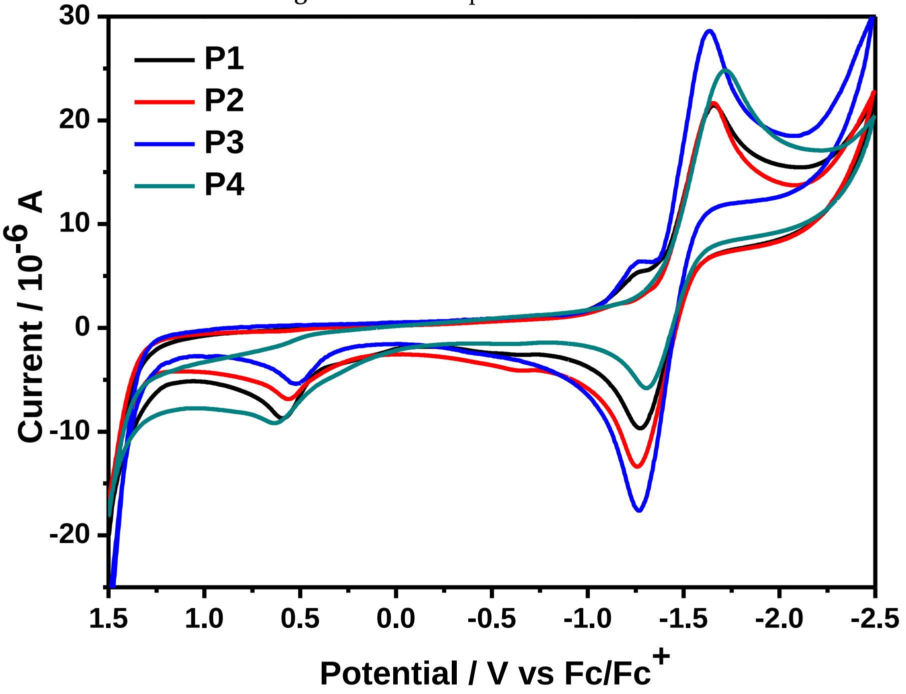

Figure S19. Cyclic voltammograms of the polymers (P1, P2, P3 and P4) in THF solutions of 0.1 mol L^{-1} Bu_4NPF_6 with a scan rate of 100 mV s^{-1}.

Table S1. Experimental and calculated electrochemical data of polymers.

Compound	HOMO (eV)	LUMO (eV)	E_g (eV)	$HOMO^{cal}$ (eV)	$LUMO^{cal}$ (eV)	E_g^{cal} (eV)
**P1**	-5.22	-3.36	1.86	-5.19	-2.85	2.35
**P2**	-5.08	-3.41	1.67	-5.15	-3.03	2.12
**P3**	-5.14	-3.25	1.89	-5.10	-2.57	2.53
**P4**	-5.11	-3.35	1.75	-5.15	-2.77	2.37

Spectroscopic responses of polymers to anions:

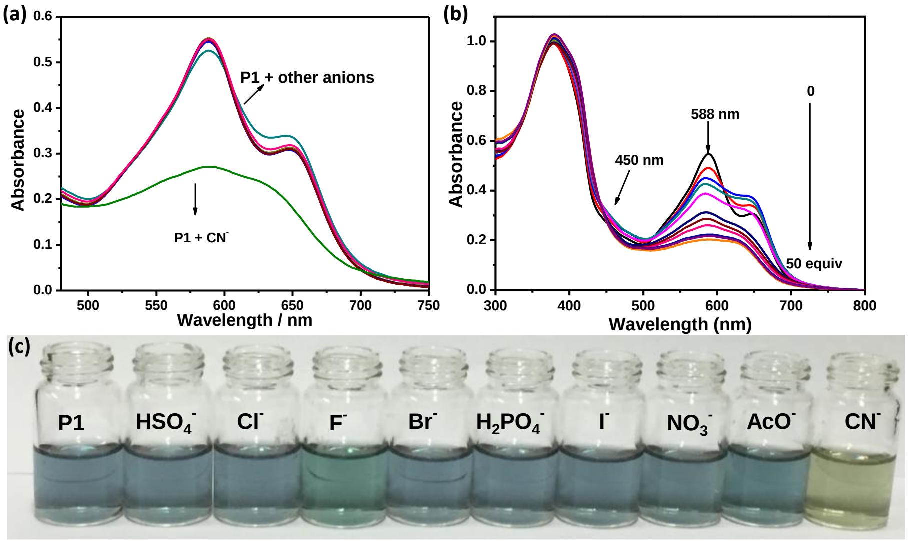

Figure S20. (**a**) UV-vis spectra of P1 ([RU] = 30μM) in THF/H2O (98:2, *v/v*) in the presence of 20 equiv. of different anions; (**b**) UV-vis spectra of P1 ([RU] = 30 μM) in THF/H2O (98:2, *v/v*) in presence of varying concentration of CN^- ranging from 0, 60, 120, 150, 210, 240, 270, 300, 450, 600, 900, 1200 and 1500 μM; (**c**) Visual images observed for P1 solutions upon addition of 50 equiv. each anion.

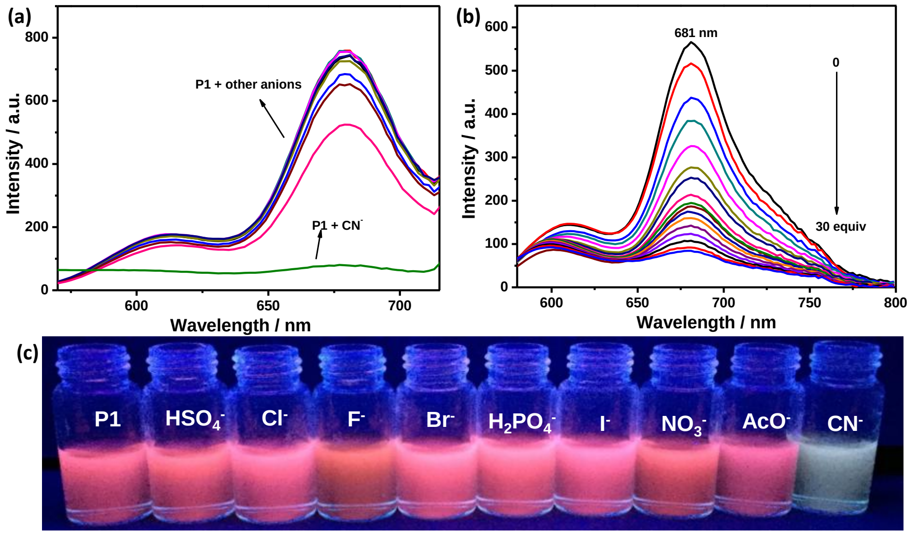

Figure S21. (**a**) Fluorescence emission spectra of P1 ([RU] = 30 μM) in THF/H2O (98:2, *v/v*) in presence of varying concentration of CN^-ranging from 0, 30, 60, 90, 120, 150, 180, 240, 300, 360, 420, 480, 540, 600, 700, 800 and 900 μM, λ_{ex} = 365 nm; (**b**) Fluorescence emission spectra of P1 ([RU] = 30 μM) in THF/H2O (98:2, *v/v*) in the presence of 20 equiv. of different anions, λ_{ex} = 540 nm; (**c**) Fluorescence observed for P1 solutions upon addition of 50 equiv. each anion under the excitation of 365 nm.

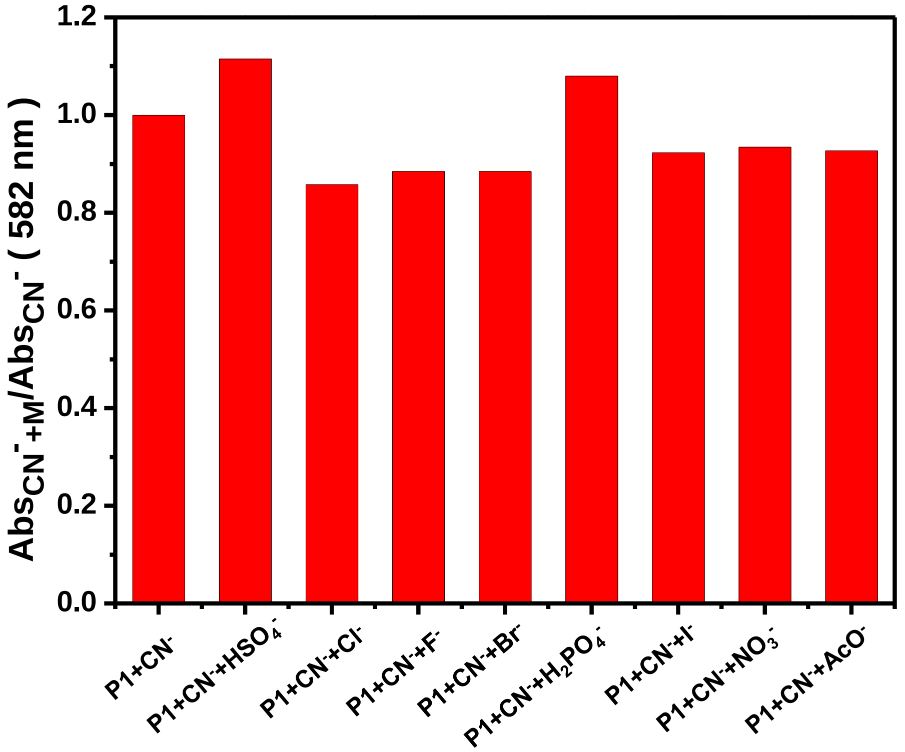

Figure S22. UV-Vis responses of P1 ([RU] = 30 μM) containing 20 equiv. of CN^- upon addition of 20 equiv. of other competing ions in THF/H_2O (98:2, *v*/*v*).

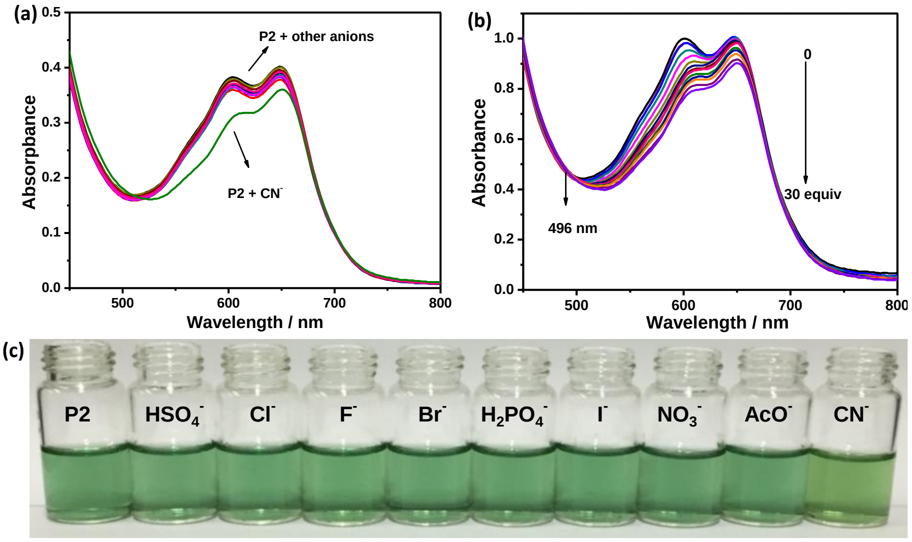

Figure S23. (**a**) UV-vis spectra of P2 ([RU] = 30 μM) in THF/H_2O (98:2, *v*/*v*) in the presence of 20 equiv. of different anions; (**b**) UV-vis spectra of P2 ([RU] = 30 μM) in THF/H_2O (98:2, *v*/*v*) in presence of varying concentration of CN^- ranging from 0, 60, 120, 180, 240, 300,360, 420, 480, 540, 600, 700, 800 and 900 μM; (**c**) Visual images observed for P2 solutions upon addition of 50 equiv. each anion.

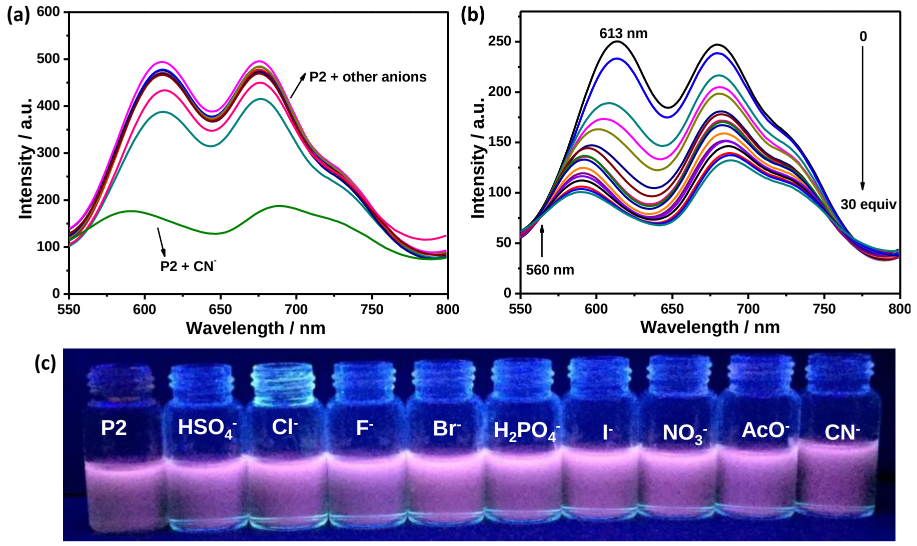

Figure S24. (**a**) Fluorescence emission spectra of P2 ([RU] = 30 μM) in THF/H_2O (98:2, *v*/*v*) in presence of varying concentration of CN^- ranging from 0, 60, 120, 180, 240, 300, 360, 420, 480, 540, 600, 700, 800 and 900 μM, λ_{ex} = 540 nm; (**b**) Fluorescence emission spectra of P2 ([RU] = 30 μM) in THF/H_2O (98:2, *v*/*v*) in the presence of 20 equiv. of different anions. λ_{ex} = 540 nm; (**c**) Fluorescence observed for P2 solutions upon addition of 50 equiv. each anion under the excitation of 365 nm.

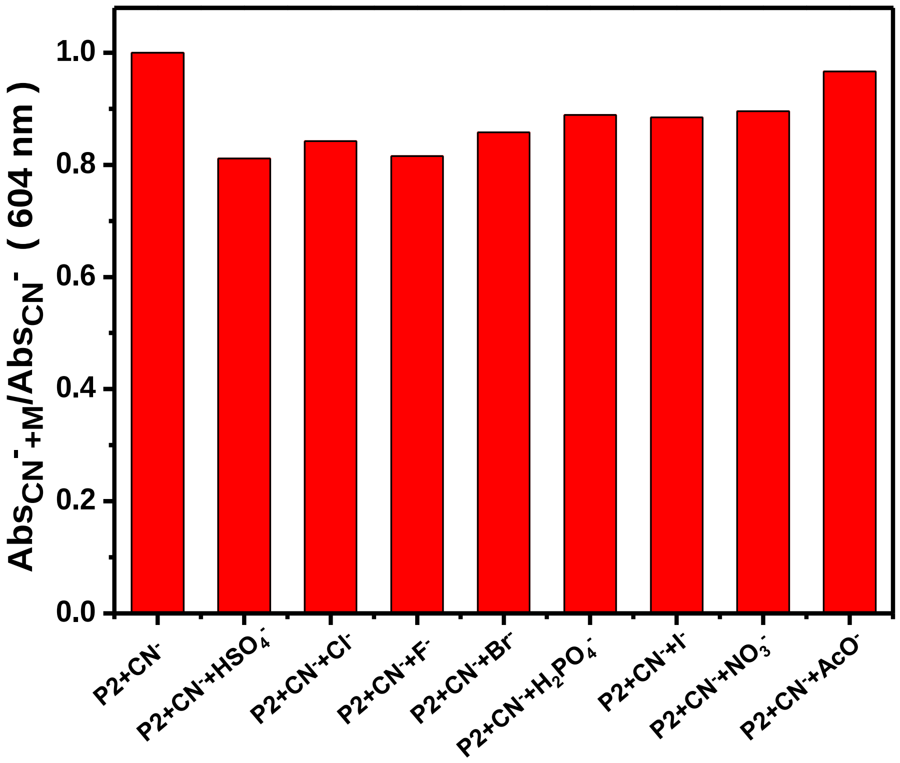

Figure S25. UV-Vis responses of P2 ([RU] = 30 μM) containing 20 equiv. of CN^- upon addition of 20 equiv. of other competing ions in THF/H_2O (98:2, *v*/*v*).

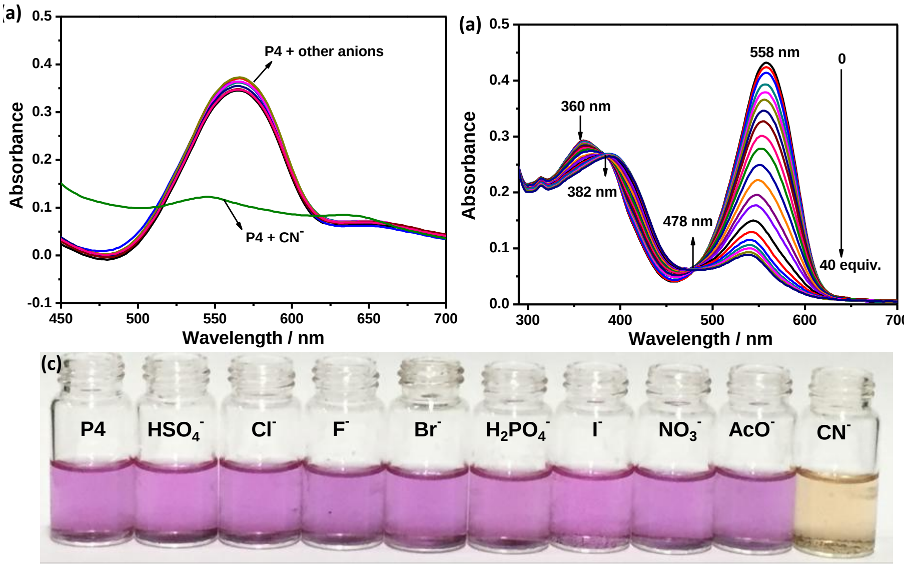

Figure S26. (**a**) UV-vis spectra of P4 ([RU] = 30 μM) in THF/H_2O (98:2, *v*/*v*) in the presence of 20 equiv. of different anions; (**b**) UV-vis spectra of P4 ([RU] = 30 μM) in THF/H_2O (98:2, *v*/*v*) in presence of varying concentration of CN^- ranging from 0, 60, 120, 150, 210, 240, 270, 300, 450, 600, 900, and 1200 μM; (**c**) Visual images observed for P4 solutions upon addition of 50 equiv. each anion.

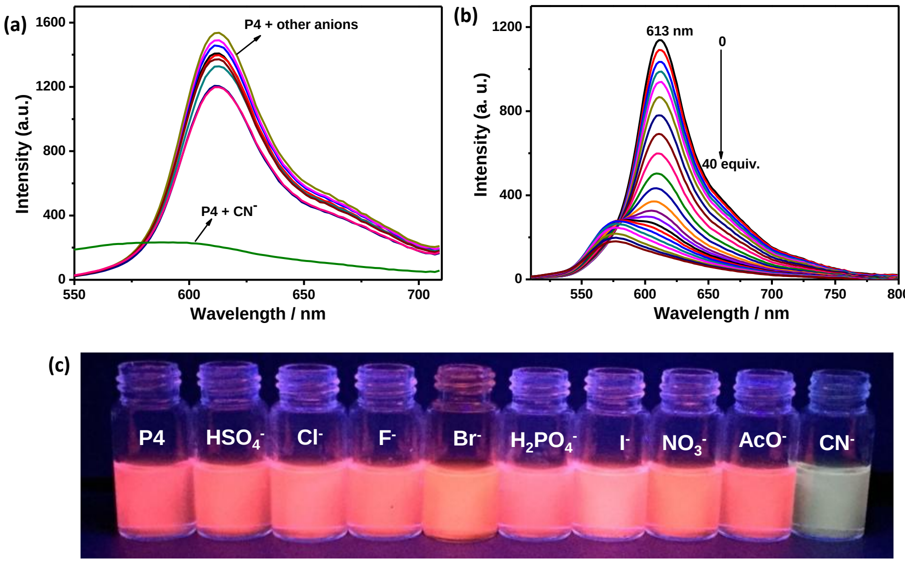

Figure S27. (**a**) Fluorescence emission spectra of P4 ([RU] = 30 μM) in THF/H_2O (98:2, *v*/*v* in the presence of 20 equiv. of different anions, λ_{ex} = 540 nm; (**b**) Fluorescence emission spectra of P4 ([RU] = 30 μM) in THF/H_2O (98:2, *v*/*v*) in presence of varying concentration of CN^- ranging from 0, 60, 120, 150, 210, 240, 270, 300, 450, 600, 900, 1200 and 1500 μM, λ_{ex} = 540 nm; (**c**) Fluorescence observed for P4 solutions upon addition of 50 equiv. each anion under the excitation of 365 nm.

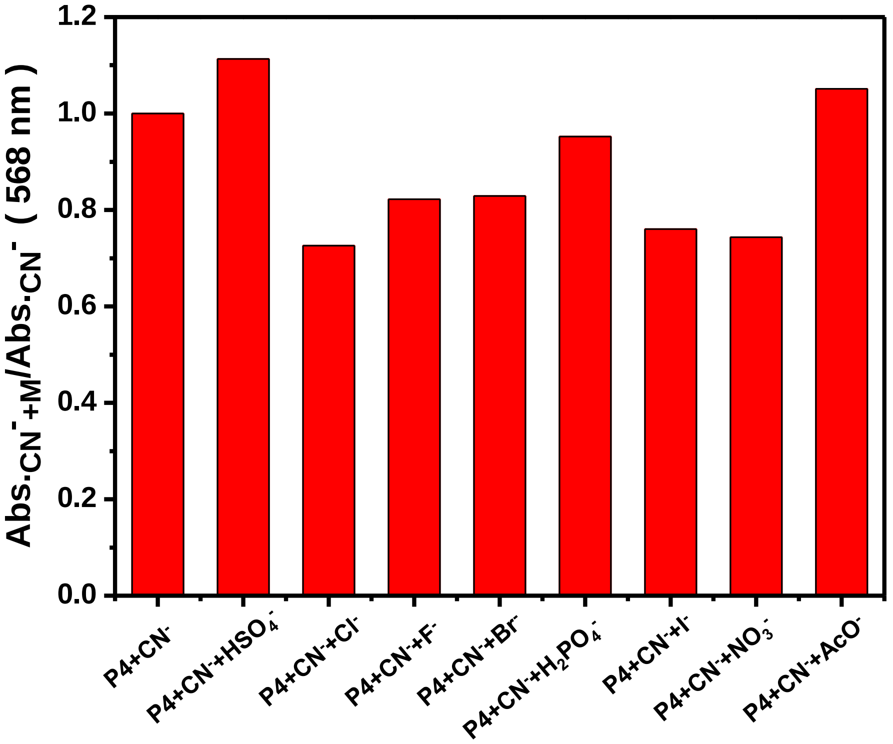

Figure S28. UV-Vis absorption changes of P4 ([RU] = 30 μM) containing 20 equiv. of CN^- upon addition of 20 equiv. of other competing ions in THF/H_2O (98:2, *v*/*v*).

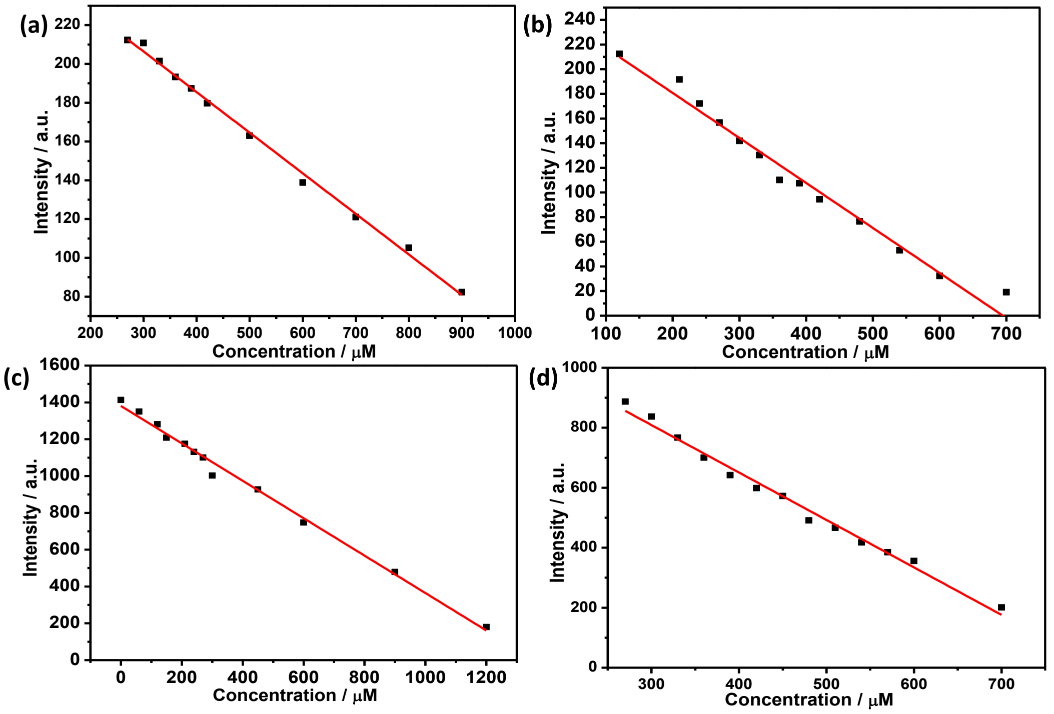

Figure S29. Plot of fluorescence intensity changes of polymers versus various concentrations of CN^- anions. The red solid lines represent the linear fitting of the scattered data points. (**a**) P1, the slope is 0.21; (**b**) P2, the slope is 0.37; (**c**) P3, the slope is 1.02; (**d**) P4, the slope is 1.58.

polymers

MDPI

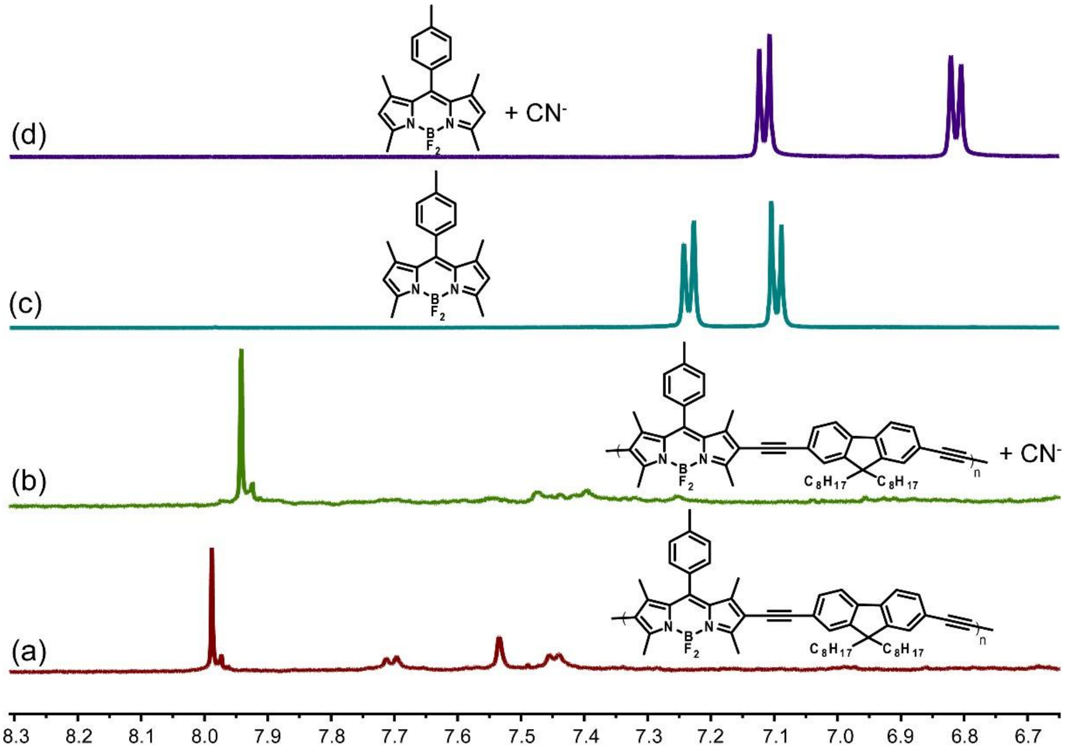

Figure S30. Partial ^{1}H NMR spectra of P3 (**a, b**) and BODIPY monomer (**c, d**) before and after the addition of 20 equiv. $[(n\text{-}Bu)_4N]^+CN^-$ (recorded in THF-d_8 at room temperature).

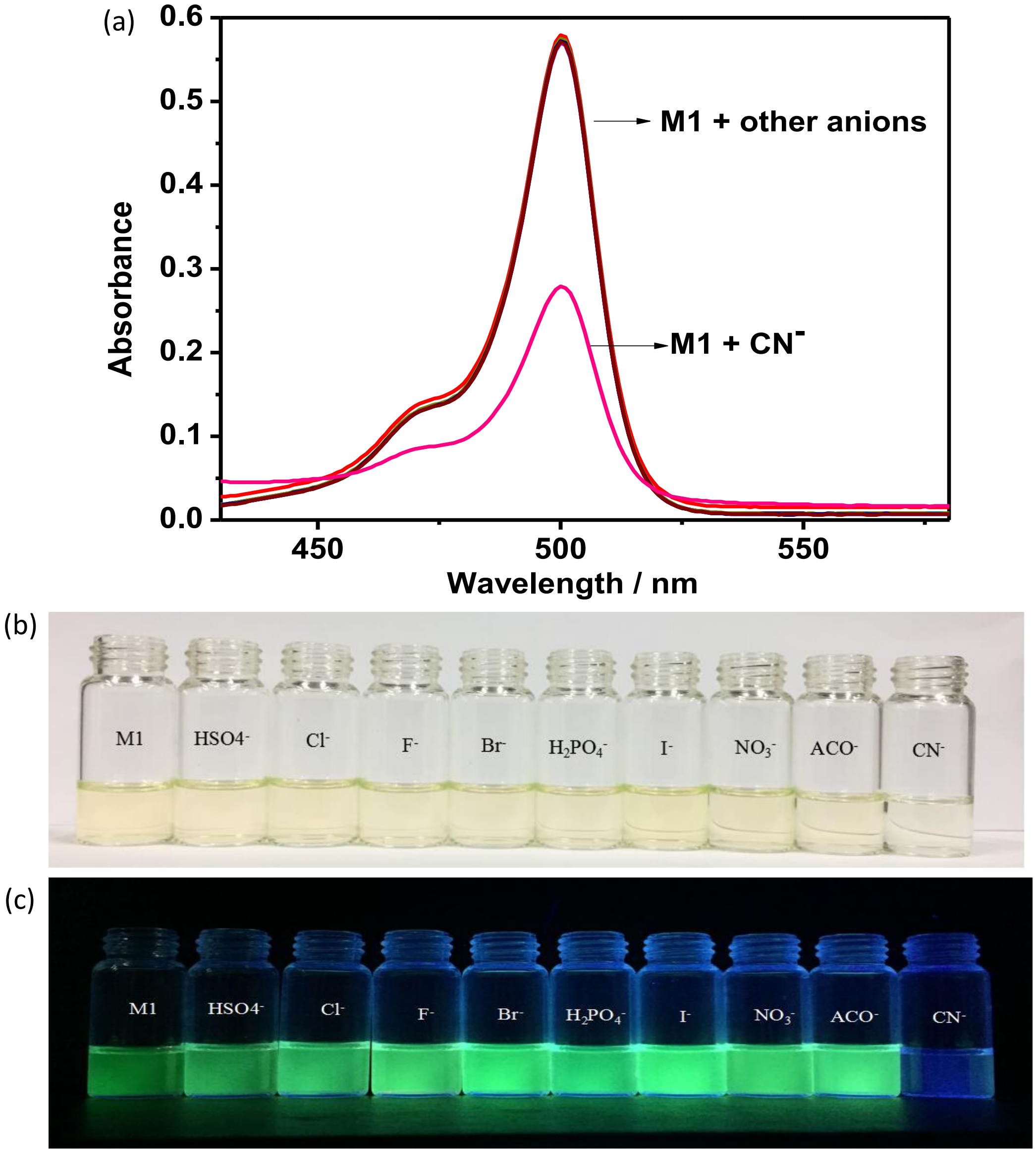

Figure S31. (**a**) UV-Vis spectra of BODIPY monomer ([RU] = 30 μM) in THF/H2O (98:2, *v*/*v*) in the presence of 20 equiv. of different anions; (**b**) Visual images and (**c**) fluorescence observed for BODIPY monomer solutions upon addition of 50 equiv. each anion under the excitation of 365 nm.
